# Expression, purification, and characterisation of the p53 binding domain of Retinoblastoma binding protein 6 (RBBP6)

**DOI:** 10.1371/journal.pone.0277478

**Published:** 2023-02-10

**Authors:** Bonnie L. Russell, Monde Ntwasa

**Affiliations:** 1 Department of Life and Consumer Sciences, College of Agriculture and Environmental Sciences, University of South Africa, Florida, Roodepoort, South Africa; 2 Innovation Hub, Buboo (Pty) Ltd, Pretoria, South Africa; University of Colorado Anschutz Medical Campus, UNITED STATES

## Abstract

RBBP6 is a 250 kDa eukaryotic protein known to be a negative regulator of p53 and essential for embryonic development. Furthermore, RBBP6 is a critical element in carcinogenesis and has been identified as a potential biomarker for certain cancers. RBBP6’s ability to interact with p53 and cause its degradation makes it a potential drug target in cancer therapy. Therefore, a better understating of the p53 binding domain of RBBP6 is needed. This study presents a three-part purification protocol for the polyhistidine-tagged p53 binding domain of RBBP6, expressed in *Escherichia coli* bacterial cells. The purified recombinant domain was shown to have structure and is functional as it could bind endogenous p53. We characterized it using clear native PAGE and far-UV CD and found that it exists in a single form, most likely monomer. We predict that its secondary structure is predominantly random coil with 19% alpha-helices, 9% beta-strand and 14% turns. When we exposed the recombinant domain to increasing temperature or known denaturants, our investigation suggested that the domain undergoes relatively small structural changes, especially with increased temperature. Moreover, we notice a high percentage recovery after returning the domain close to starting conditions. The outcome of this study is a pure, stable, and functional recombinant RBBP6-p53BD that is primarily intrinsically disordered.

## 1. Introduction

Worldwide, cancer is a significant health issue. Cancer is caused by a cascade of events that result in abnormal cell proliferation due to defective cell cycle progression [1, 2]. The *p53* gene is a crucial tumour suppressor gene that encodes the protein Tp53, which, amongst other functions, initiates cell cycle arrest in response to DNA damage or apoptosis if DNA is beyond repair. MDM2 is a negative regulator of p53 that binds to p53 and causes p53’s ubiquitination and, therefore proteasome degradation [3, 4]. The mouse orthologue of RBBP6 interacts with p53, and this interaction appears to enhance MDM2-mediated ubiquitination and proteasome degradation of p53 within the cells [3, 5, 6]. Levels of p53 are strictly controlled within the cell by an auto-regulatory feedback loop, as MDM2 is transcriptionally activated by p53, and MDM2 results in p53’s degradation [7–9].

The Retinoblastoma binding protein 6 (RBBP6), a 250kDa eukaryotic protein, has been linked to many biological processes, including transcription, mRNA metabolism, embryonic development, and ubiquitination [6, 10–12]. RBBP6 consists of several highly conserved domains, including an N-terminal ubiquitin-like domain (Domain with no name (DWNN)), a CCHC zinc finger domain, a RING (Really Interesting New Gene) finger domain, a proline-rich domain, a serine/arginine (S-R) domain, as well as p53 and pRB binding domains. RBBP6 is overexpressed in several cancers and has been identified as a potential cancer biomarker [13–17]. This, therefore, makes RBBP6 a candidate protein for cancer therapy for cancers where native p53 is maintained [18].

With RBBP6’s part in facilitating p53’s degradation within the cell, a need for understanding its mechanism of interaction with p53 has arisen. Research has been conducted to define the residues of RBBP6 which correspond to the p53 binding domain of RBBP6. Simons and colleagues assigned the p53 binding domain in PACT to residues 1220–1562, corresponding to residues 1428–1792 in full-length RBBP6 [10]. Later, Gao and Scott assigned it to 110 amino acids, namely residues 1204–1314 of P2D-R, corresponding to residues 1380–1490 in RBBP6 [19].

In their post-graduate studies, Ndabambi and Faro independently investigated the p53 binding domain of RBBP6 by expressing different peptide fragments fused with GST and GST-HA tags in bacterial cells [20, 21]. Ndabambi analysed the complete sequence predicted by Simons and colleagues and found the domain unstable and prone to proteolysis [20]. Faro found the residue sequence 1422–1668 of full-length RBBP6 could bind to p53, despite NMR analysis suggesting it was completely unfolded [21]. Furthermore, this study found that these residues are unstable and prone to proteolysis. Thus far, we are not aware of any successful recombinant expression of a stable and functional RBBP6-p53BD.

Twala performed *in silico* analysis on residues 1433–1544 from the UniprotKB database (Unique entry identifier: Q7Z6E9) entry of RBBP6 [22]. To predict the structure of the domain from the amino acid sequence, two software programs were used, namely I-TASSER and eThread-Modeller. It was found that the top predicted models from both programs contained predominantly alpha helices and some random coil. These models were further used to predict potential drugs that could bind the domain, and NADH was identified. It is noteworthy that Twala’s study did not include the entire RBBP6-p53BD as experimentally defined in various studies.

To target the interaction between RBBP6 and p53, a better understanding is needed of the p53 binding domain of RBBP6, its structure, and its ability to interact with p53. This present study expressed and investigated an insert covering residues 1380–1726 of full-length human RBBP6, named RBBP6-p53BD going forward in this article. The aim of this study was to determine if a stable and functional RBBP6-p53BD could be expressed in a bacterial cell system, successfully purified and probed for structure and stability.

## 2. Methods and materials

### 2.1 Materials

See Table 1 for the antibodies used in the study.

**Table 1:** Antibodies used in the study.

Antigen	Antibody	Use	Dilution	Species	Company	Product code
P53	Monoclonal IgG2a	WB	1:10000	Mouse	Abcam	ab1101
		IP	1:500			
Polyhistidine	Monoclonal IgG	WB	1:5000	Mouse	Sigma	H-1029
		IP	1:250			
Mouse	Polyclonal	WB	1:10000	Goat	Abcam	ab205719

### 2.2 Expression of the p53 binding domain of RBBP6

A pET28a plasmid was engineered to contain the cDNA sequence encoding the p53 binding domain of RBBP6 and procured from GenScript^®^ (Shanghai, China). For the insert, residues 1380–1726 of full-length RBBP6 (NM_006910.4) were used. NiCo21 (DE3), BL21(DE3), and Shuffle^®^ T7 express *Escherichia coli* cells were transformed, and single colonies were grown for 16 hours in sterile fresh LB media inoculated with 30mg/ml kanamycin, before a 50 times dilution (1:50 dilution) was prepared into fresh LB media containing kanamycin (30mg/ml). The cell cultures were grown to mid-log (calculated using optical density) and induced with between 0.1mM and 1.0mM IPTG and grown for 2–16 hours post-induction, at 37°C.

Samples were then centrifuged for 20 minutes at room temperature, suspended in buffer, and sonicated, at 60 Amps for 6 cycles of 10 seconds (2 seconds on 1 second off), on ice. The sonicated samples were then centrifuged for 20 minutes at 12,100 x g at 4°C to analyse soluble and insoluble fractions. Samples collected were analysed using SDS-PAGE.

### 2.3 Western blot

Proteins were separated using SDS-PAGE and transferred onto polyvinylidene difluoride (PVDF) membranes before being blocked with casein blocking buffer (Sigma, B6429). Membranes were then incubated in primary antibodies overnight at 4°C, before being incubated with secondary antibodies for 1 hour 30 minutes, at room temperature. Membranes were washed with Tris-buffered saline with 0.1% Tween (TBST) after antibody incubations.

Proteins were visualised with SuperSignal^™^ West Pico PLUS Chemiluminescent Substrate (Thermo Scientific, Massachusetts, United States) and photographed using a BioRad, Universal Hood III (Hercules, USA) gel imaging system.

### 2.4 Purification of RBBP6-p53BD

For purification, the cell pellet from a larger volume of LB was sonicated for 16 cycles of 30 seconds each (2 seconds on, 1 second off), at 80 Amps. A three-part purification protocol was undertaken. All purifications were performed using a Biorad NGC^™^ Chromatography Quest 10 system (cat# 788–0001) and a PC with ChromLab software installed (Hercules, USA).

For part one, the supernatant of NiCo21 (DE3) cells was incubated with 1.0 M ammonium sulphate for 30 minutes before being centrifuged at 4,400 rpm for 20 minutes and being loaded onto a Cytiva, Phenyl high-performance 5ml column. The column was equilibrated with binding buffer (50 mM sodium phosphate with 1.0 M ammonium sulphate, pH 7). After the sample was loaded, the column was washed with 10 column volumes of binding buffer, before the sample was eluted off the column by decreasing the ammonium sulphate concentration in a gradient from 1.0 M to 0 M. The flow-through and fractions that showed a high A280nm absorbance was analysed using SDS-PAGE. The RBBP6-p53BD was found not to bind the column and instead was found in the flow-through.

For part two, the flow-through collected from part one was incubated with 1.4 M ammonium sulphate for 30 minutes before being centrifuged at 4,400 rpm for 20 minutes and being loaded onto a Cytiva, Phenyl high-performance 5ml column. The column was equilibrated with binding buffer (50 mM sodium phosphate with 1.4 M ammonium sulphate, pH 7). After the sample was loaded, the column was washed with 10 column volumes of binding buffer, before the sample was eluted off the column by decreasing ammonium sulphate concentration in a gradient from 1.4 M to 0 M. The flow-through and fractions that showed a high A280 nm absorbance was analysed using SDS-PAGE. The RBBP6-p53BD was found to bind the column and be eluted off the column along with several non-specific bacterial proteins.

For part three, the fractions identified to contain the RBBP6-p53BD from part two were combined and dialysed into 50 mM sodium phosphate with 500 mM sodium chloride and 40 mM imidazole, pH 7.4. Dialysis was performed with three buffer changes with a 4–18 hour incubation for each change at 4°C, and a buffer volume 10 times the sample size. The dialysed protein sample was then loaded onto a Cytiva Ni-Sepharose 5ml high-performance column that had been equilibrated with binding buffer (50 mM sodium phosphate with 500 mM sodium chloride and 40 mM imidazole, pH 7.4). Proteins were eluted off the column by increasing imidazole concentration, in a gradient from 40 mM to 500 mM. The flow-through and fractions that showed a high A280nm absorbance were analysed using SDS-PAGE.

### 2.5 Mass spectrometry

Partially purified recombinant RBBP6-p53BD with polyhistidine tag was analysed using SDS-PAGE. The bands of interest were excised and sent to CSIR (Pretoria, South Africa) for trypsin digested liquid chromatography-mass spectrometry (LC-MS) analysis [23, 24].

### 2.6 Native PAGE

Two types of clear native PAGEs were performed. Firstly, a continuous 8% Tris-HCl gel was used, with a running buffer of 25 mM Tris with 192 mM glycine, pH 8.3. Secondly, an 8% continuous gel was used as described in [25], was performed using an imidazole-HEPES buffer system at a pH of 7.4. The RBBP6-p53BD has a predicted pI of 9, by ProtParam tool on the ExPASy proteomics server [26] (S1 Fig), which resulted in the electrodes needing to be reversed. The gels were run at 140 volts for two hours before being stained with Coomassie Brilliant blue stain and visualised using a Biorad Universal hood lll (Hercules, USA).

### 2.7 Spectroscopy

Far-UV Circular Dichroism (CD) spectra measurements were performed between 250 nm and 190 nm, depending on the sample. A 1 mm quartz cuvette was used. The step size between measurements was 1nm, the bandwidth was 1 nm, and a scan speed of 2 nm/second was used. Data obtained was converted to mean residue ellipticity. An Applied Photophysics Chirascan Plus instrument (Leatherhead, England), equipped with a Peltier temperature controller was used. Buffer contributions were subtracted for all measurements. Three repeats were made for each sample and averaged. In addition, experimental conditions were repeated at least twice to confirm reproducibility. The concentration of protein was determined using a fluorescent Qubit^®^ Protein Assay Kit and the Qubit^®^ 2.0 Fluorometer (Thermo Scientific, Waltham, Massachusetts, USA).

### 2.8 Thermal denaturation of RBBP6-p53BD

Far-UV CD measurements were collected as described in section 2.7, whilst heating the protein sample from 20°C to 90°C. A spectrum was recorded at two-degree increments and a final spectrum once the protein was cooled back to 20°C.

### 2.9 Chemical denaturation of RBBP6-p53BD

The stability of the RBBP6-p53BD was tested in the presence of urea of guanidinium chloride. Guanidinium chloride and urea were prepared using the necessary buffer as the solvent and the pH was adjusted as needed before being filtered. Stock solution concentrations were confirmed using an Atago R5000 Refractometer (Tokyo, Japan), using refractive indices described by [27]. Samples were incubated in buffers containing the desired denaturant concentration for 1 hour before measuring. Recovery studies were undertaken by diluting the protein back down from 6 M to 1 M for guanidinium chloride and 8M to 0.5M for urea. Protein samples were incubated for one hour at each diluted concentration before spectra were recorded. Spectra were recorded at 22°C as described in section 2.7.

### 2.10 Co-immunoprecipitation

HEK293T cells were kindly donated from the National Institute for Occupational Health (NIOH) by Dr Jitcy Joseph. HEK293 cells are human embryonic kidney cells which were transfected with DNA from adenovirus 5. HEK293T cells are a clone derivative of HEK293, which express the large T antigen of simian virus 40 (SV40). HEK293T cells were lysed by sonication for 5 cycles of 10 seconds at 60 Amps while cells were kept on ice. Lysed cells were then centrifuged at 12,100 x g for 20 minutes at 4°C before 50μl/ml of protease inhibitor was added. The purified, recombinant RBBP6-p53BD was added to the cell lysate and the mixture was incubated at 4°C with rotation for 3 hours. Next, the primary antibody was added to the cell lysate and the recombinant RBBP6-p53BD mixture and was incubated for 3 hours at 4°C with rotation. Finally, protein A agarose was added to the cell lysate/antibody/recombinant protein mixture and incubated for another 12–16 hours at 4°C with rotation.

Unbound protein was removed, and the beads were washed 5 times with PBS and centrifuged at 1000 x g for 5 minutes. As the antibody heavy chain is at a similar molecular weight as p53, we performed two elution steps in series in samples from anti-p53 Co-IP assays. Firstly, the beads were boiled in sample buffer without the presence of a reducing agent (β-mercaptoethanol) to preserve the antibody structure. After centrifugation, the supernatant was removed and the beads were next boiled in SDS-PAGE sample buffer with a reducing agent. Both elution steps were analysed using SDS-PAGE and Western blot. For the anti-polyhistidine Co-IP assays, a single elution step was performed where the beads were boiled in SDS-PAGE sample with a reducing agent. Samples were then analysed using SDS-PAGE and Western blot (section 2.3).

## 3. Results

### 3.1 Recombinant expression of the p53 binding domain RBBP6 (RBBP6-p53BD)

Mammalian proteins are notoriously different to express in bacterial cell lies. Therefore, the expression of recombinant RBBP6-p53BD, was investigated in three *Escherichia coli* strains, namely BL21 (DE3), NiCo21 (DE3), and Shuffle^®^ T7 express, under varying conditions. To determine the best expression system the three cell lines were BL21 (DE3), which is one of the most commonly used *E*.*coli* cell lines, with minimal genetic manipulation. BL21 (DE3) and NiCo21 (DE3) cells contain the λDE3 lysogen, in which theT7 RNA polymerase gene is controlled by an IPTG inducible lac UVS promoter [28]. NiCo21 (DE3) cells were chosen as they have been genetically modified to reduce non-specific protein binding during Nickel IMAC purification [29]. SHuffle^®^ T7 Express cells are genetically engineered to have an altered redox state that allows for the formation of stable disulphide bonds within the bacterial cell cytoplasm and express DsbC in the cytoplasm, which assists the cells to isomerise mis-oxidized protein to their native states. This is because DsbC is an oxidoreductase chaperone capable of assisting in the oxidative folding of proteins [30]. Even though RBBP6 p53BD does not contain a disulphide bond (it only has a single cysteine residue), some proteins incorrectly folded or expressed insolubly, fold correctly when expressed in SHuffle^®^ T7 Express cells [31].

**Fig 1:**
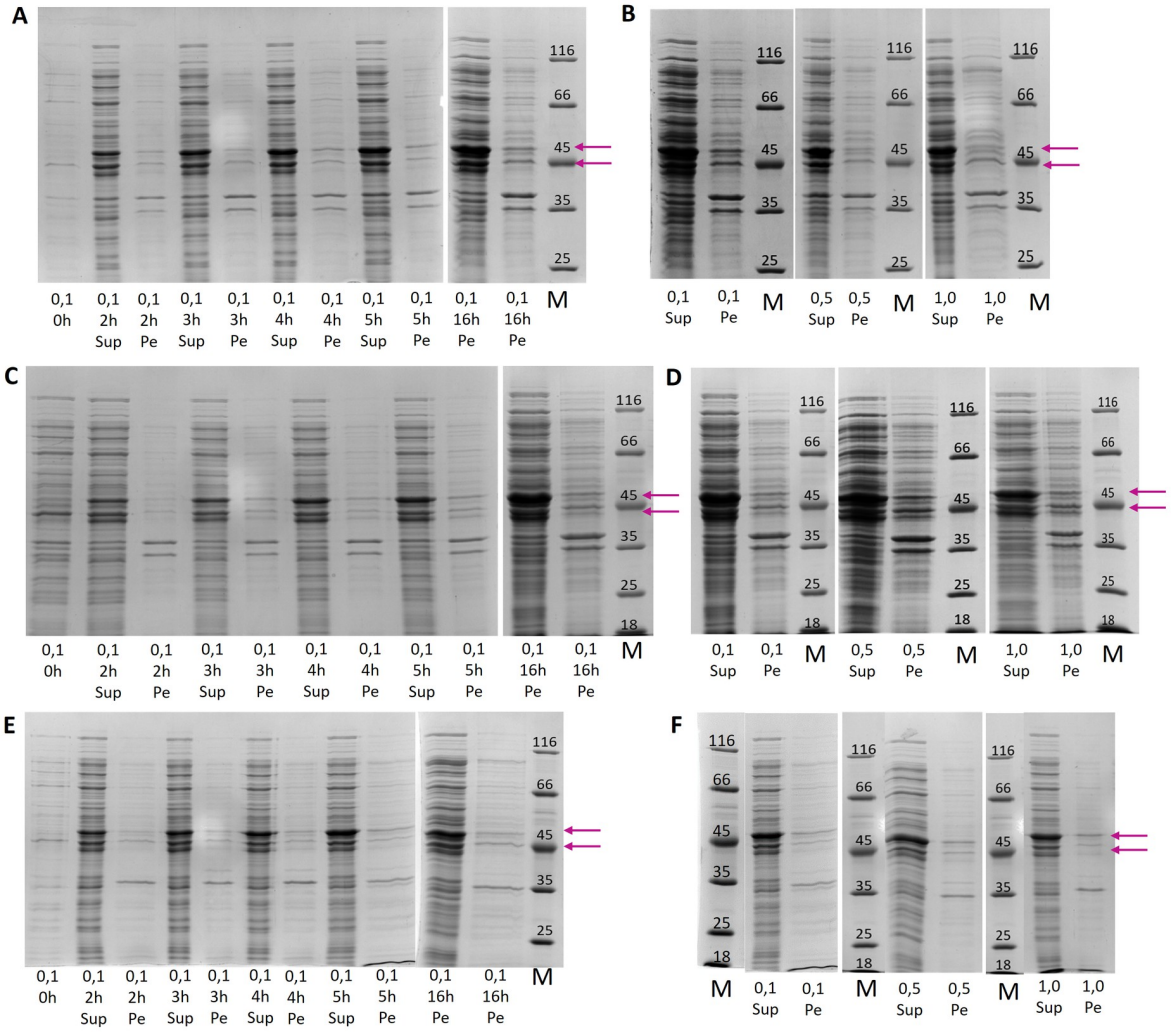
Recombinant expression of RBBP6 p53BD. SDS-PAGE analysis of expression of the RBBP6-p53BD showing soluble "sup" and insoluble "Pe" fractions for **(A)**: BL21 (DE3) cells over post-induction time of 2 to 16 hours, **(B)**: BL21 (DE3) cells inducer concentrations from 0.1 mM to 1.0 mM, **(C)**: NiCo21 (DE3) cells over post-induction time of 2 to 16 hours, **(D)**: NiCo21 (DE3) cells inducer concentrations from 0.1mM to 1.0mM. **(E)**: Shuffle^®^ T7 express cells over post-induction time of 2 to 16 hours, **(F)**: Shuffle^®^ T7 express inducer concentrations from 0.1 mM to 1.0 mM. The position of overexpressed protein bands is marked with pink arrows. The protein molecular weight marker (Thermo Scientific, 26610) has sizes in kDa, marked on the gel.

In all cell lines, a post-induction time was evaluated between 2 and 16 hours, and three inducer concentrations were evaluated, namely 0.1 mM, 0.5 mM, and 1.0 mM. All experiments were conducted at 37°C. With SDS-PAGE analysis, two prominent protein bands were seen in the soluble fraction with estimated molecular weights of 48kDa and 44kDa in all cell lines (Fig 1), which is not the anticipated 40kDa of the RBBP6-p53BD. However, there are several reasons why a protein may migrate at an unexpected rate through SDS-PAGE, including the content of the primary sequence, presence of hydrophobic regions, SDS interaction, and protein structure and stability [32–34].

Expression of the RBBP6-p53BD in BL21 (DE3) and NiCo21 (DE3) cells was found to be similar, with noticeably less expression seen in Shuffle^®^ T7 express cells. In all three cell lines, the RBBP6-p53BD appears to be predominantly in the soluble fraction, and there was an increase in expression from 2 to 16 hours (Fig 1A, 1C and 1E). We noted that there was little change in expression with an increase in inducer concentration in any of the cell lines (Fig 1B, 1D and 1F). Higher inducer concentrations are known to place increased stress on cells during expression. NiCo21 (DE3) cells have been genetically engineered to express fewer proteins that contain polyhistidine residues that can interact with IMAC columns [29]. This reduces the isolation of non-recombinant proteins during the purification process. Therefore, expression conditions chosen for future studies were NiCo21 (DE3) cells at 37°C, with 0.1 mM IPTG and 16 hours post-induction growth.

### 3.2 Protein confirmation

**Fig 2:**
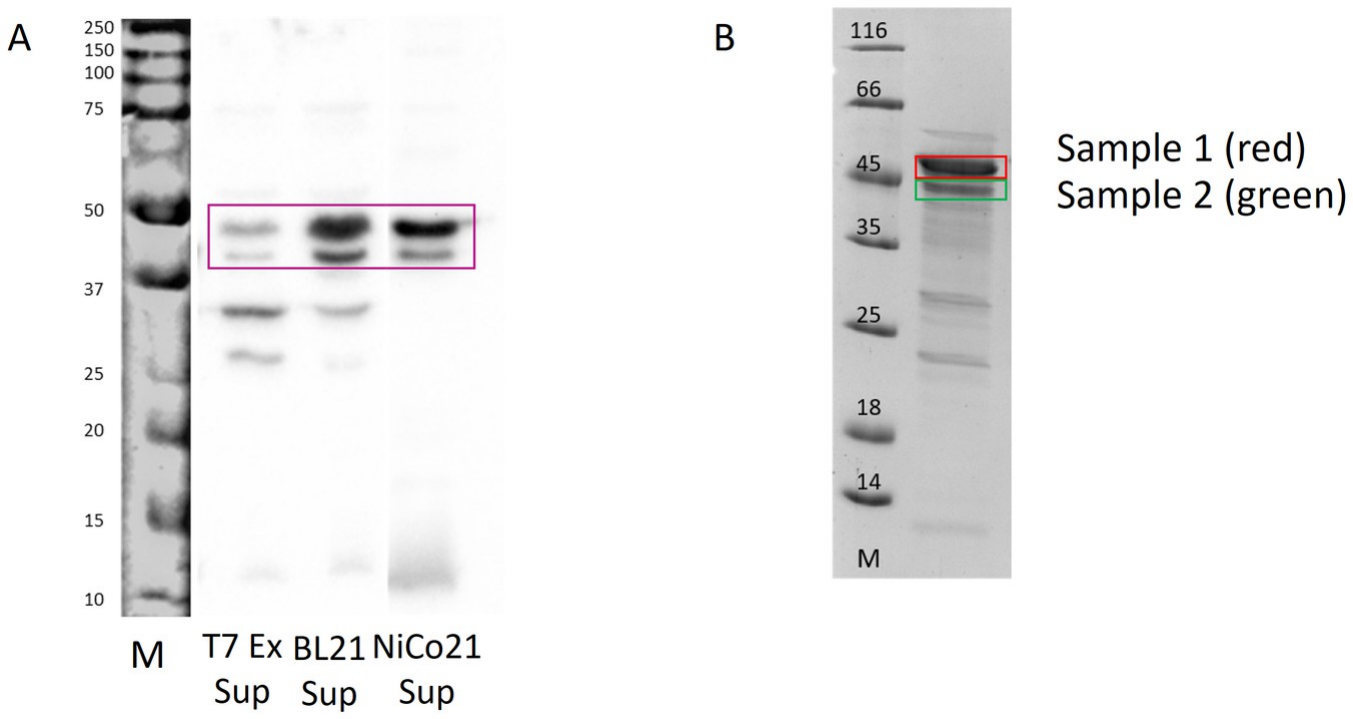
Protein conformation of RBBP6-p53BD. **(A)**: Shows western blot analysis of supernatant fractions of cell lysate from transformed Shuffle^®^ T7 express, BL21 (DE3), and NiCo21 (DE3) E.coli cell lines using an anti-polyhistidine antibody. The RBBP6-p53BD is shown with a pink box. **(B)**: After SDS-PAGE the two protein bands indicated (red and green boxes) were isolated for trypsin digested LC-MS analysis after partial purification of the supernatant from NiCo21 (DE3) cells. The protein molecular weight marker (Thermo Scientific, 26610) has sizes in kDa, marked on the gel.

We needed to investigate whether both protein bands seen in SDS-PAGE analysis after purification, contained RBBP6-p53BD. We achieved this by performing western blot analysis and trypsin digested LC-MS analysis. Western blot analysis was undertaken using an anti-polyhistidine antibody (Fig 2A) on the soluble fraction of cell lysates from Shuffle^®^ T7 express, BL21 (DE3), and NiCO21 (DE3) *E*.*Coli* cell lines. It showed that both overexpressed protein bands contained polyhistidine residues. This suggests these bands are the RBBP6-p53BD with a polyhistidine tag. This is significant as the protein contains a polyhistidine tag on its C-terminal and therefore, only full length recombinant RBBP6-53BD would be able to bind the nickel IMAC column [35]. However, naturally occurring proteins in bacteria cells can also contain polyhistidine residues that anti-polyhistidine antibodies can detect. Therefore, the two protein bands were further analysed using trypsin digested LC-MS analysis (Fig 2B). The bands were confirmed to contain the RBBP6-p53BD. Thus, further investigation could be performed using the purified sample containing both bands.

### 3.3 Three-part purification protocol for RBBP6-p53BD

**Fig 3:**
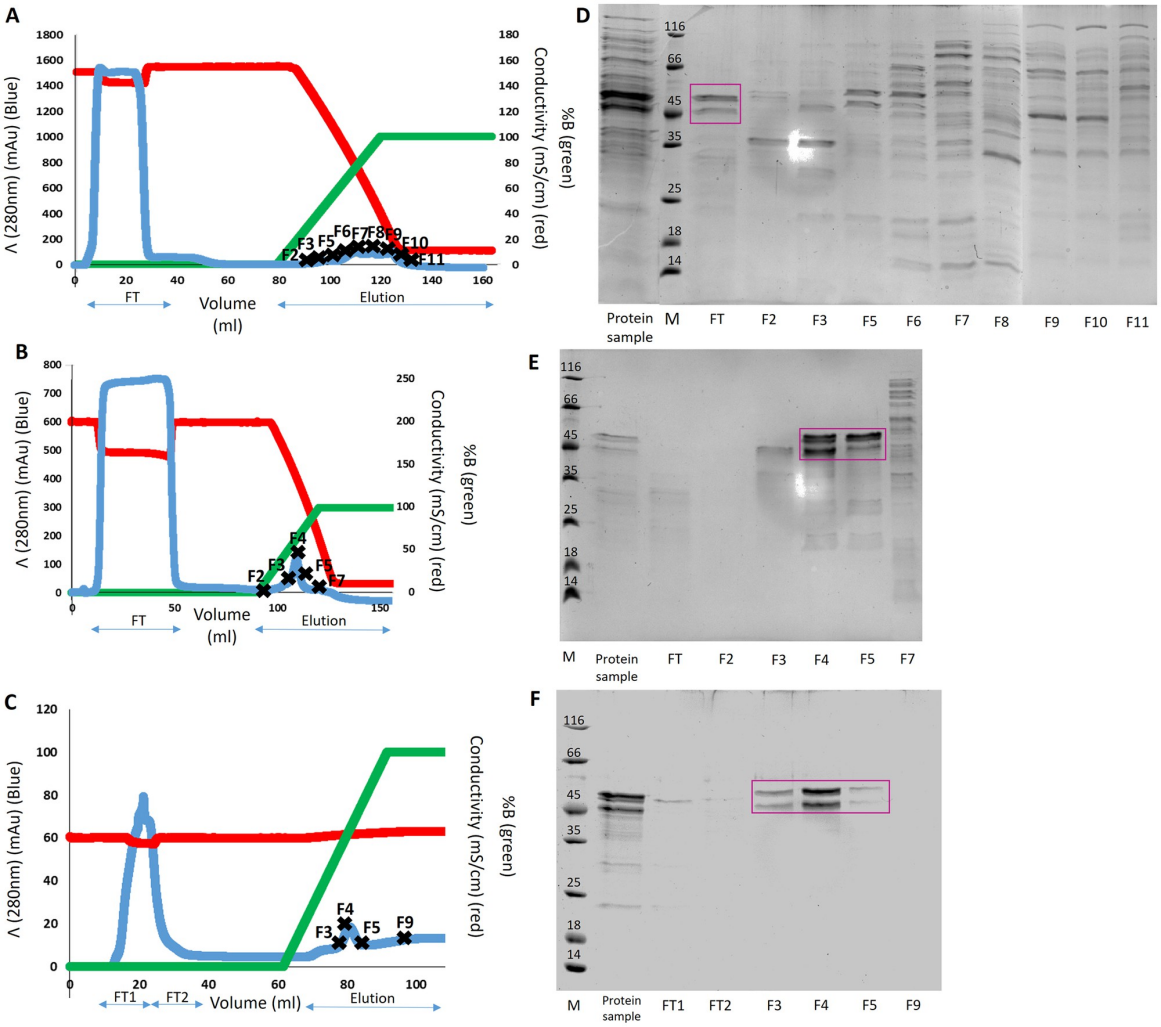
Three-part purification of the RBBP6-p53BD. Chromatograph with A280 effluent (blue), elution gradient (green), conductivity (red) and Flow-through (FT), elution and fractions collected (FX) depicted for **(A)**: Part 1, hydrophobic interaction chromatography with 1 M ammonium sulphate in binding buffer **(B)**: Part 2 hydrophobic interaction chromatography with 1.4 M ammonium sulphate in binding buffer and **(C)**: Part 3, nickel IMAC chromatography. SDS-PAGE analysis of protein sample loaded onto the column, the Flow-through (FT), and certain fractions collected "FX" where X represents the fraction number for **(D)**: Part 1, **(E)**: Part 2, and **(F)**: Part 3. The RBBP6-p53BD is indicated in flow-through (FT) or fractions (FX) with a pink box. The protein molecular weight marker (Thermo Scientific, 26610) has sizes in kDa, marked on the gel.

To isolate RBBP6-p53BD out of the bacterial cell lysate it was necessary to perform three protein purification protocols in series. In part one, the soluble fraction of NiCo21 (DE3) cells were prepared in 1.0 M ammonium sulphate before being loaded onto an equilibrated Cytiva HiTrap Phenyl high performance (Uppsala, Sweden) column. Proteins were eluted off the column through a 1 M to 0 M ammonium sulphate gradient (Fig 3A). Fractions with high A280 absorbance readings were analysed with SDS-PAGE (Fig 3D). The RBBP6-p53BD did not bind to the column and was instead found in the flow-through (FT). However, multiple non-specific bacterial cellular proteins could bind the column and were eluted under these conditions, thereby removing them from the protein sample.

In part two, the flow-through from part one was incubated in 1.4 M ammonium sulphate before being loaded onto an equilibrated Cytiva HiTrap Phenyl high performance (Uppsala, Sweden) column. Proteins were eluted off the column by decreasing the ammonium sulphate concentration in a gradient from 1.4 M to 0 M (Fig 3B). The RBBP6-p53BD bound the column and was eluted predominately in fractions 4 and 5, where the ammonium sulphate concentration had decreased to approximately 0.85 M (Fig 3E). Fractions with high A_280_ absorbance readings were again analysed by SDS-PAGE (Fig 3E).

Dialysis was performed with three buffer changes with a 4–18 hour incubation for each change at 4°C, with buffer volume 10 times the sample size. Several bacterial cell proteins bound the column and were eluted under the same conditions as the RBBP6-p53BD in part two. Therefore, for part three the fractions 4 and 5 from part two were dialysed into 50 mM sodium phosphate with 500 mM sodium chloride and 40 mM imidazole, pH 7.4. The dialysed protein sample was then loaded onto Cytiva HisTrap high performance (Uppsala, Sweden) column. Proteins were eluted off the column by increasing the imidazole concentration from 40 mM to 500 mM using a gradient (Fig 3C), and fractions with high A280 absorbance readings were analysed with SDS-PAGE (Fig 3F). The RBBP6-p53BD was found to bind the column and was eluted off in fractions 3, 4, and 5 (Fig 3F). The fractions containing the RBBP6-p53BD were then pooled and concentrated. The RBBP6-p53BD was estimated to be approximately 95% pure when using BioRad Image Lab^™^ software version 6.0.1 (Hercules, USA) "Relative Quantity" tool.

### 3.4 Physicochemical and structural characterization

#### 3.4.1 Clear native PAGE

**Fig 4:**
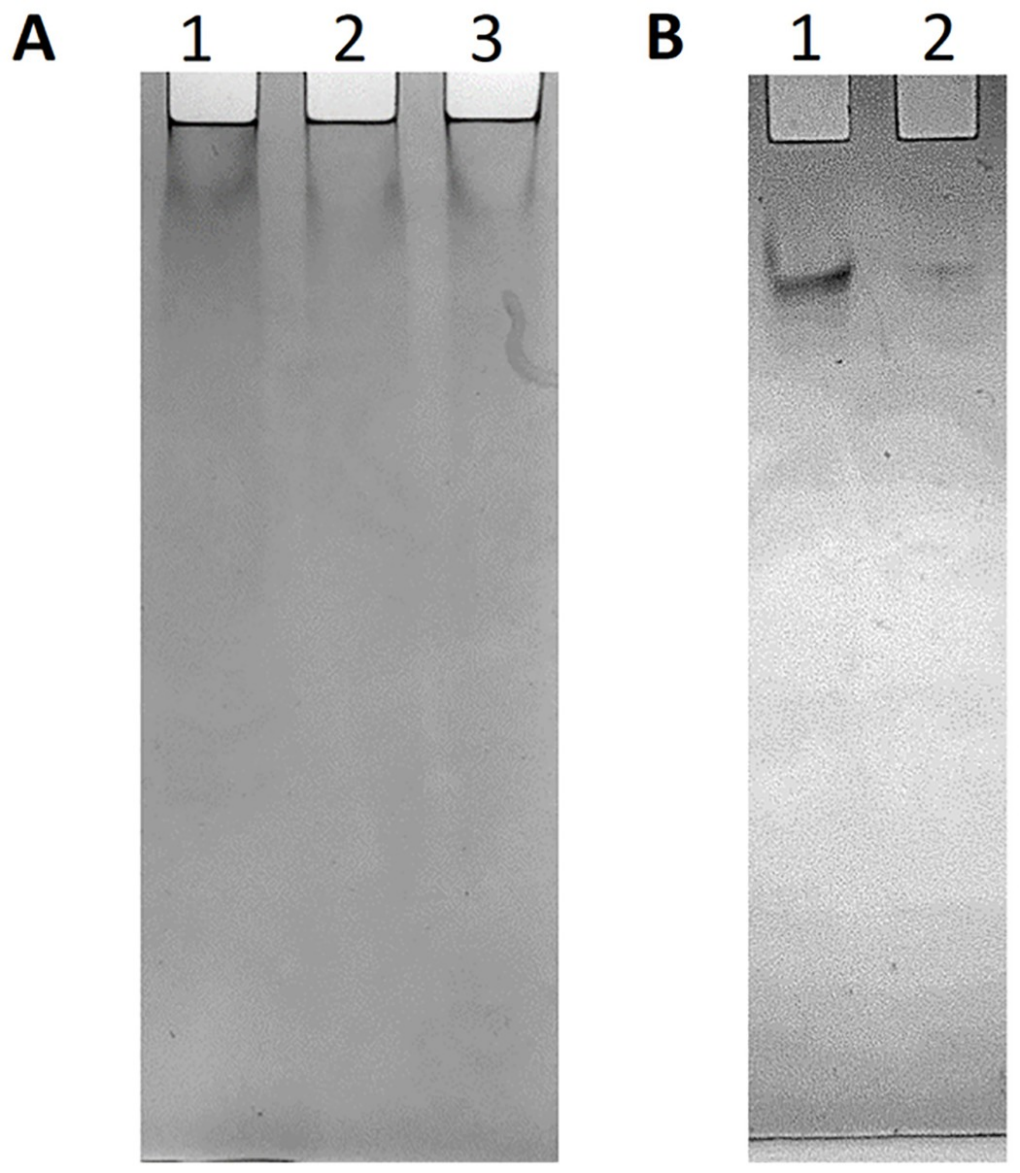
Clear native PAGE analysis of the RBBP6-p53BD. Clear native PAGE analysis of purified RBBP6-p53BD. **(A)**: Tris-HCl buffer, with pH 8.3. Lane 1 is the RBBP6-p53BD in 50 mM sodium phosphate, pH 7. Lane 2 is the RBBP6-p53BD in 50 mM sodium phosphate, pH 7 after being heated to and cooled from 90°C to 20°C, as described in **section 2.8**. Lane 3 is the RBBP6-p53BD in 50 mM sodium phosphate, pH 7 with 2 M urea. **(B)**: Imidazole-HEPES buffer, pH7.4. Lane 1 is the RBBP6-p53BD in 50 mM sodium phosphate, pH 7, and Lane 2 is the RBBP6-p53BD in 50 mM sodium phosphate, pH 7 after being heated to and cooled from 90°C to 20°C, as described in **section 2.8**. Protein samples were not equalised before loading onto the gels.

Clear native PAGE separates proteins based on their size, charge and 3-D structure, as it’s preformed under non-denaturing conditions. Clear native PAGE is used to investigate the presence of multimeric forms of a protein. Although oligomerisation is frequently important for a protein’s structure and function it can complicate the characterisation of a protein. Therefore, it is beneficial to determine if a protein is naturally a monomer or oligomer before further characterisation. We investigated two buffer systems for the RBBP6-p53BD. Firstly we used a Tris-HCl system with a pH of 8.3 (Fig 4A), which showed that the RBBP6-p53BD appears in a single form both before (lane 1) and after (lane 2) being heated to 90°C. Lastly, in lane 3 we showed a single form when the domain was placed in 2 M urea. However, some protein appears to still be present in the gel wells and as the pH of the Tris-HCl buffer was close to the pI of the RBBP6-p53BD (pI of 9) there was little migration into the gel. Therefore, we performed a second clear native PAGE using an imidazole-HEPES buffer system with a pH of 7.4 (Fig 4B), which showed that the RBBP6-p53BD appears to be in a single form before (lane 1) and after being heated to 90°C and cooled (lane 2). However, a greater migration into the gel is seen at the lower pH. We could not demonstrate the precise oligomer state of the RBBP6-p53BD using this technique as the molecular weight cannot be determined using a clear native PAGE. However, the results suggest that the domain is in monomer form as even in 2 M urea or after being heated, a single band is seen at the same distance migrated as the protein without denaturants.

#### 3.4.2 Secondary structure characterization

**Fig 5:**
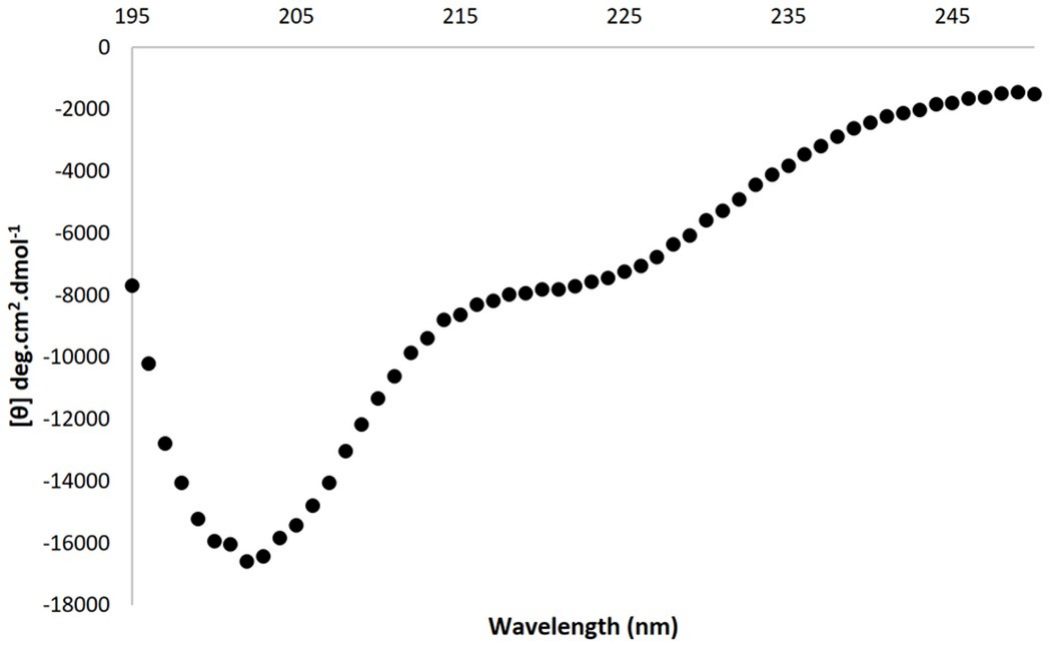
Far-UV CD spectrum of the RBBP6-p53BD. Far UV CD spectra of the RBBP6-p53BD, showing 250 nm to 195 nm for 2.6 μM RBBP6-p53BD in 20 mM sodium phosphate, pH 7.

The spectrum produced by far-UV CD can be used to access a protein’s secondary structure, as different protein conformations, such as alpha helices and beta strands, produce characteristic features in the spectrum. Far-UV circular dichroism was utilised to investigate the secondary structure of the purified RBBP6-p53BD. The spectrum for the RBBP6-p53BD shown in Fig 5, was recorded from 250 nm to 195 nm as the noise to signal ratio was too high below 195 nm, as detected by the dynode voltage.

Using DICHOWEB [36], the secondary structure of the RBBP6-p53BD was predicted using the far-UV CD spectrum. Three programs within DICHOWEB, namely CONTIN, SELCON3, and CDSSTR [36] were used. Several references sets were utilised to produce percentage predictions of alpha-helices, beta-strands, turns, and random coils (Table 2). Reference set 7 showed the lowest NRMSD score in all three programs. From the three programs, using reference set 7, we obtained an averaged prediction of 19% alpha-helical, 9% beta strands, 14% turns, and 60% random coil. The NRMSD value indicates how accurate the prediction is, and ordinarily, a score below 0.1 is considered a good fit. However, SELCON3 is known to have a higher score on average and a score below 0.5 has been seen as acceptable. For the CDSSTR program, reference sets sp175 and 4 had acceptable NRMSD scores and their predictions showed higher structural content, particularly a much higher prediction of beta strands than reference set 7.

**Table 2:** DICHROWEB secondary structure predictions.

Program	Reference set used	NRMSD	% alpha helix	% beta strands	% turns	% coil
CONTIN	sp175	0,71	21	16	16	46
CONTIN	7	0,097	20	3	12	65
CONTIN	4	0,117	18	16	27	36
SELCON3	sp175	0,604	22	18	17	44
SELCON3	7	0,442	16	14	15	60
SELCON3	4	0,604	24	16	25	34
CDSSTR	sp175	0,019	23	17	18	42
CDSSTR	7	0,014	21	10	14	55
CDSSTR	4	0,019	18	19	26	34

DICHROWEB [36] predicted secondary structure components using three different software server programs: CONTIN, SELCON3, and CDSSTR. The reference set used in each prediction is indicated in the table. NRMSD is a score of how accurate the prediction is.

#### 3.4.3 Computational protein structure prediction

**Fig 6:**
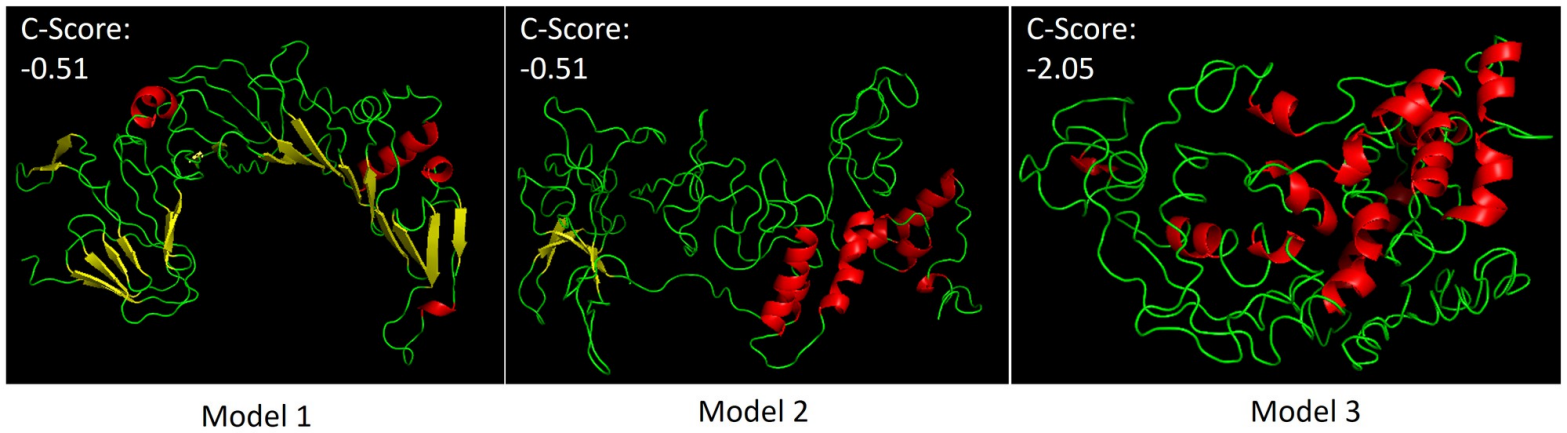
I-TASSER software prediction of secondary and tertiary structure. Top three 3D conformation models predicted by I–TASSER, with predicted c-Score shown on each model. Alpha-helices are shown in red, beta-strands in yellow, and loops shown in green. The structure was obtained from the protein data bank (PDB) files produced by I-TASSER and viewed with PyMOL Molecular Graphics System (2.5.2 Edu), Schrödinger, LLC.

I-TASSER is a program that predicts a protein’s structure by identifying structural templates from the PDB databank library using a multiple threading alignment approach called LOMETS [37]. The top three, 3D models representing the RBBP6-p53BD’s structural conformation produced by LOMETS in I-TASSER software are shown in Fig 6. It was predicted that the RBBP6-p53BD consists predominantly of random coil, with alpha-helical and beta-strand structures varying from model to model (Fig 6). The confidence of each model is measured using the c-score and is shown on each model in Fig 6. The c-score is generated based on the significance of the treading template alignment and convergence of parameters of the structure assembly simulations. The acceptable range for a c-score is -5 to 2, with a higher score signifying a model with higher confidence; however, a score greater than -1.5 indicates a model of correct global topology [38]. Global accuracy prediction is done using c-score and protein length for model 1 by I-TASSER and consists of a TM-Score and RMSD value. For model 1 the estimated TM-score was 0.65±0.13, and the estimated RMSD was found to be 10.9±4.6Å. A TM-score is found to be between 0 and 1, with a TM greater than 0.5 indicating a model of correct topology. Models 2 and 3 generally have lower TM scores compared to model 1 and therefore aren’t reported by I-TASSER. Therefore, based on I-TASSER’s parameters, Model 1, is the most likely model for the RBBP6-p53BD.

A secondary structure server, namely 2Struc [39] predicted the percentage of alpha-helices and beta-strands for the RBBP6-p53BD models generated by I-TASSER, using the protein data bank files generated by I-TASSER (Table 3). Percentages of alpha-helices, beta-strands, and others (a mixture of turns and random coil) varied appreciably from model to model, with the highest alpha-helical content found in model 3 and highest beta-strand prediction found in model 1. Model 2 showed the highest random coil percentage prediction and three times the alpha-helical content compared to its beta-strand content (Table 3). However, when comparing the models to the prediction made by DichroWeb [36], it is seen that model 2 has a similar ratio of alpha-helices to beta-strands as seen in predictions made from the far-UV CD spectrum. However, I-TASSER did predict a lower percentage of structure overall, with 14% alpha-helices to 4% beta-strand for model 2 compared to an average of 19% alpha-helices to 9% beta-strand from the far-UV CD spectrum using DichroWeb.

**Table 3:** Prediction of alpha-helical and beta-strand protein content for I-TASSER predicted structures of RBBP6 p53BD.

Model	Program	% Alpha helix	% beta strands	%Other
Model 1	DSSP	12,9	28,6	58,5
	STRIDE	10,5	28,3	61,2
Model 2	DSSP	14,4	3,9	81,6
	STRIDE	14,2	5,2	80,6
Model 3	DSSP	25,2	0	74,8
	STRIDE	26,5	1,0	72,4

2Struc secondary structure server predicated alpha-helical and beta-strand content of RBBP6 p53BD for the three top models predicted by I-TASSER software [39].

The amino acid composition of a protein can indicate the likelihood of intrinsic order or disorder. Order-promoting amino acids (Trp, Met, Cys, Phe, Ile, Tyr, Val, Leu and Asn) are frequently found in folded globular proteins. In contrast, a high percentage of polar and charged amino acids (Gln, Ser, Pro, Glu, Lys, Gly, Ala and Arg), are known to indicate disorder and are overrepresented in unstructured proteins [40, 41]. RBBP6 p53BD sequence investigated in this study comprises of 63.6% amino acid residues that promote disorder and only 19.8% amino acid residues that promote order (S1 Fig) [26].

### 3.5 Conformational stability

#### 3.5.1 Thermal-induced unfolding

**Fig 7:**
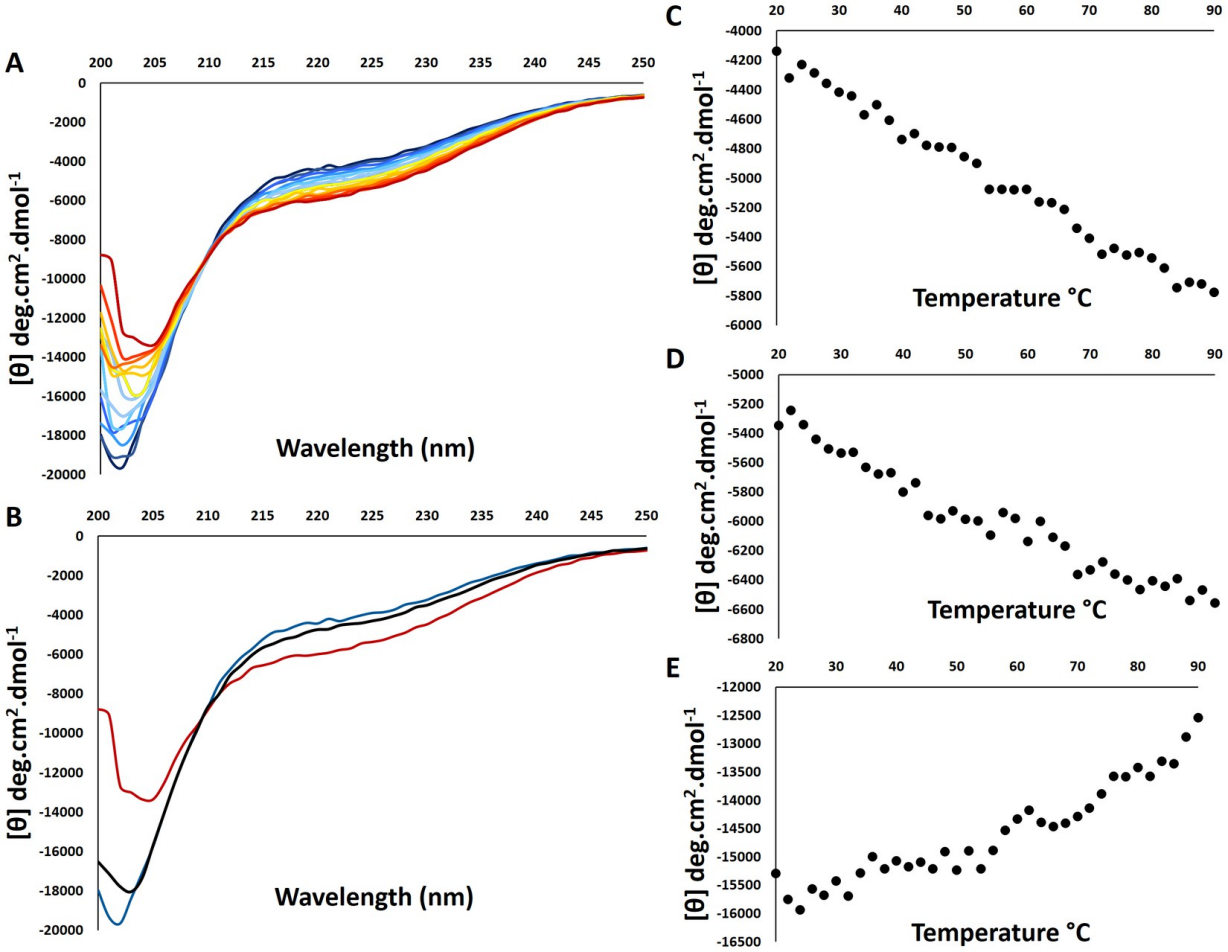
Far-UV circular dichroism monitoring of thermal unfolding of RBBP6 p53BD. **(A)**: Far UV CD spectrum for the RBBP6-p53BD from 20°C and 90°C (dark blue to dark red). **(B)**: Spectrum for the RBBP6-p53BD in the native state (blue), when heated to 90°C (red) and cooled back to 20°C (black). The heat unfolding curves for the RBBP6-p53BD at **(C)**: 222nm, **(D)**: 215nm, and **(E)**: 208nm between 20°C and 90°C.

Protein stability studies are essential for several downstream experiments, including drug interaction studies and protein crystallography. We used Far-UV CD to evaluate the thermal stability of the RBBP6-p53BD’s secondary structure. In Fig 7B we note that thermal unfolding is reversible for the RBBP6-p53BD. This is because the recovery percentage is highest at 208 nm, at 99.6%, followed by 94% at 215 nm and 91% at 222 nm. This shows an over 90% recovery at all wavelengths investigated based on the native spectrum (blue) in comparison to the spectrum heated to 90°C (red) and cooled back down to 20°C (black). Structural change percentage was found to be highest at around 222 nm, at 29%, followed by 215 nm at 19% and 208 nm with the least change of just 7%. As temperature increases, an increase in ellipticity between 200nm and 210 nm is seen (Fig 7A). However, the ellipticity between 210 nm and 250 nm decreased, showing structural changes upon heating suggestive of the protein structure becoming more compact in parts (Fig 7A). An isodichroic point is seen at 210 nm, where the signal is independent of temperature. Even at 90°C, the spectrum has not yet reached a characteristic spectrum of an unfolded protein, suggesting structure remains at 90°C [42].

Unfolding curves at 222nm (Fig 7C), 215nm (Fig 7D), and 208nm (Fig 7E), show a negative linear relationship with temperature for 222nm and 215nm across 20 to 90°C, suggesting a small consistent change in structure with an increase in temperature (Fig 7C and 7D). At 208nm (Fig 7E) the curve shows small structural changes between 20 and 50°C and more substantial changes after 50°C to 90°C.

#### 3.5.2 Characterization of the RBBP6-p53BD in the presence of denaturants

**Fig 8:**
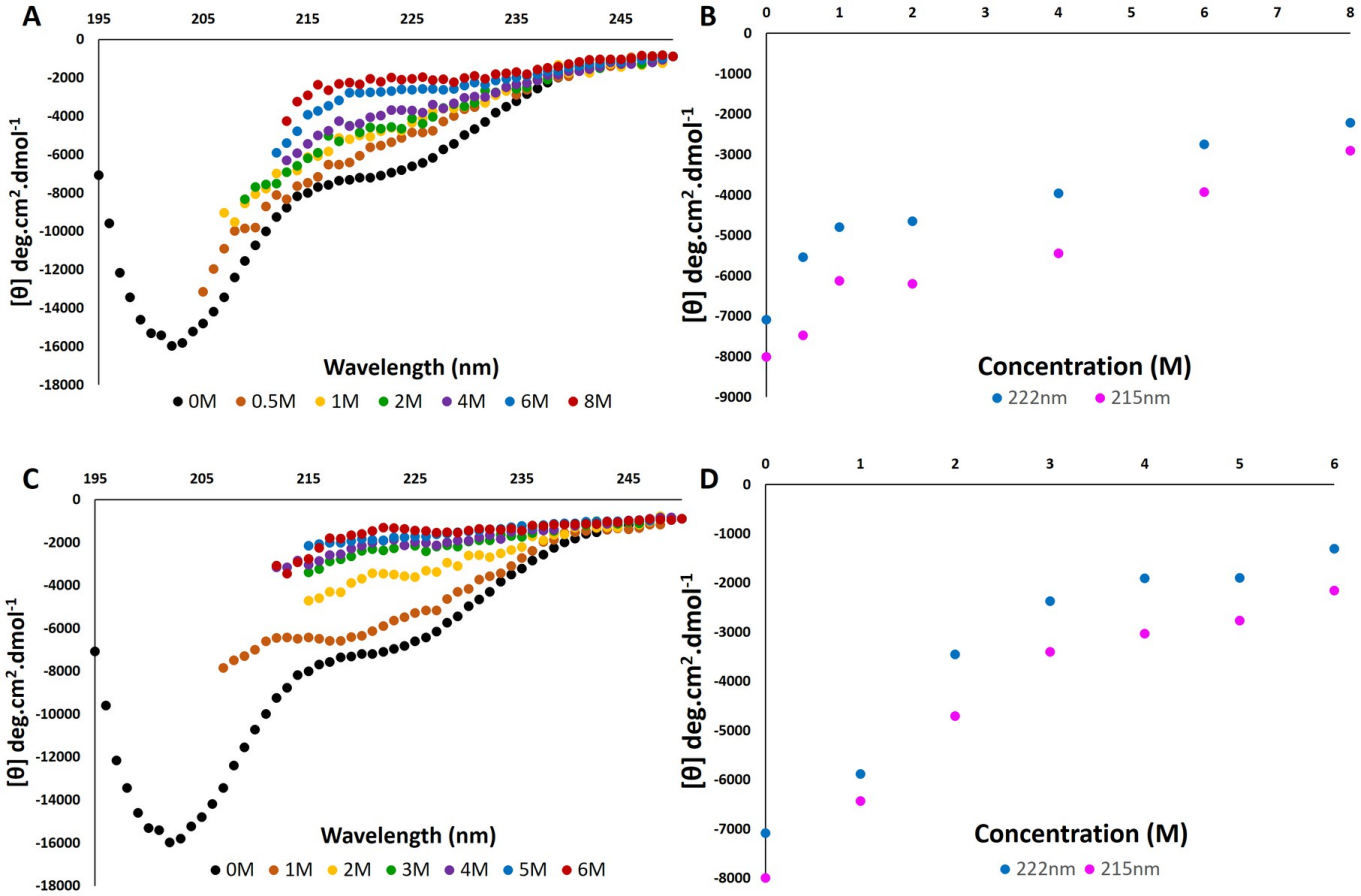
RBBP6 p53BD in the presence of denaturants. **(A)**: Far-UV CD Spectra between 250 nm and 195 nm for the RBBP6-p53BD from 0 to 8 M urea **(B)**: Far UV-CD curves at 215 nm and 222 nm for 0 to 8 M urea. **(C)**: Far-UV CD Spectra between 250 nm and 195 nm for the RBBP6-p53BD from 0 to 6M guanidinium chloride. **(D)**: Far UV-CD curves at 215 nm and 22 2nm for 0 to 6 M guanidinium chloride.

A commonly used technique for investigating protein stability is to evaluate the structure of a protein in the presence of denaturants. Denaturants are chemicals that are known to cause the unfolding of protein molecules. Far-UV CD spectra were recorded for purified RBBP6-p53BD in the presence of urea (Fig 8A) and guanidinium chloride (Fig 8C), two well-known denaturants. The RBBP6-p53BD was independently placed into 8 M urea and then diluted down to 0.5 M urea and 6 M guanidinium chloride and diluted down to 1 M respectively to investigate refolding. The far-UV CD spectra could only be recorded to, at most, 210 nm in the presence of urea and guanidinium chloride as the noise to signal ratio was too high below this wavelength, as detected by turbidity (dynode voltage).

In urea, the spectrum showed structural loss for the domain in 8 M urea and recovery of structure when diluted back to 0.5 M urea (Fig 8A). Structural change at 222 nm was found to be 69% and at 215 nm is 64%, with recovery at 222 nm being 78%, at 215 nm being 93% and at 208 nm, 80%. In Fig 8B the increase in mean residue ellipticity at both 215 nm and 222 nm is indicative of significant structural changes occurring consistently up to 8 M urea.

In guanidinium chloride structural loss was seen for the RBBP6-p53BD in 6 M guanidinium chloride and recovery of structure when diluted back to 1 M (Fig 8C). Structural change at 222 nm was found to be 74% and at 215 nm is 73%, with recovery at 22 2nm being 83%, at 215nm being 80% and at 208nm, 60%. In Fig 8D the increase in mean residue ellipticity at both 215 nm and 222 nm is indicative of significant structural changes up to 3 M, and less significant structural changes occurring from 3 M to 6 M.

### 3.6 Recombinant RBBP6 p53BD interaction with endogenous p53

**Fig 9:**
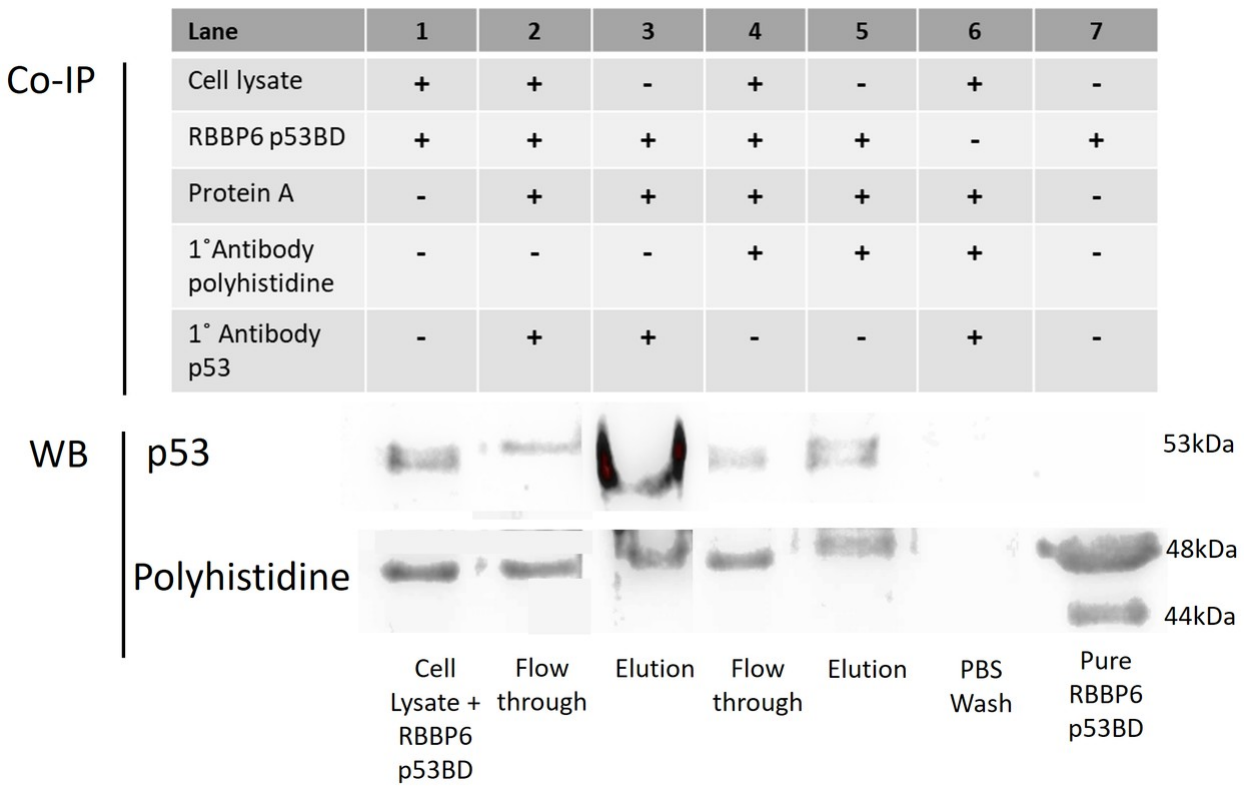
Western blot analysis of Co-IP assays performed using anti-p53 and anti-polyhistidine on HEK293 T cells. Two separate Western blots were performed to investigate the presence of endogenous p53 and recombinant polyhistidine-tagged RBBP6 p53BD in Co-IP assays performed using protein A agarose with anti-p53 (lanes 2 and 3) and anti-polyhistidine (lanes 4 and 5) as probes. Lane 1 contains the HEK293 T cells cell lysate with purified RBBP6-p53BD which was used in Co-IP assays. Lanes 2 contains the flow-through from the anti-p53 Co-IP assay which consists of the proteins in the cell lysate that did not bind with the p53 antibody. Lanes 3 contains the immuno-complexes isolated during the anti-p53 Co-IP assays. Lanes 4 contains the flow-through from the anti-polyhistidine Co-IP assay which consists of the proteins in the cell lysate that did not bind with the polyhistidine antibody. Lane 3 contains the immuno-complexes isolated during the anti-polyhistidine Co-IP assay. Lane 6 contains the PBS collected from washing the agarose beads before elution for both Co-IP assays combined. Lane 7 contains purified RBBP6-p53BD as a control. Lane 3 in both blots was overexposed, and therefore a shorter exposure time was used for this lane and overlaid on top.

Two separate Co-IP assays were performed using anti-p53 and anti-polyhistidine as the probing antibody, respectively. HEK293T cell lysate was incubated with purified, recombinant RBBP6-p53BD before incubation with antibodies. The subsequent immune-complexes formed were isolated using Protein A agarose beads. Lastly, a Western blot was used to analyse the results, as shown in Fig 9. When investigating the presence of p53 in the samples collected, we found that p53 was present in the immuno-complexes isolated from both Co-IP assays (lanes 3 and 5). We expected the presence of p53 in the anti-p53 Co-IP assay’s immuno-complexes (lane 3). Therefore, its appearance in the anti-polyhistidine Co-IP assay immuno-complexes (lane 5) suggests that endogenous p53 and recombinant RBBP6-p53BD can bind and form a complex *in vitro*. p53 was also seen in the cell lysate sample (lane 1) and the flow-through in the anti-p53 Co-IP (lane 2) and anti-polyhistidine Co-IP (lane 4), this could be due to endogenous RBBP6 being present in the HEK293T cell lysate and competing for p53 binding, resulting in not all p53 present being able to bind the recombinant RBBP6-p53BD.

The presence of the RBBP6-p53BD in the samples collected was investigated using an anti-polyhistidine antibody during western blot (Fig 9). The recombinant protein was found in the immuno-complexes isolated from both Co-IP assays (lanes 3 and 5), further supporting that endogenous p53 can bind to and form a complex with the recombinant RBBP6-p53BD. The RBBP6-p53BD was also found in the flow-through of both Co-IP assays (lanes 2 and 4), which may be due to excess recombinant RBBP6-p53BD being present to cause a competitive advantage to the domain over endogenous full-length RBBP6. As the RBBP6-p53BD was seen in the flow-through, the PBS wash step was analysed (lane 6) to confirm that the presence of the RBBP6-p53BD in the immuno-complexes samples was not just due to excess being present. Purified recombinant RBBP6-p53BD was analysed in lane 7, as a positive control for anti-polyhistidine Western blot and as a negative control to show that anti-p53 antibodies do not interact directly with the recombinant RBBP6-p53BD. The ability of the purified RBBP6-p53BD to bind to endogenous p53 suggests that the domain is folded and functional.

## 4. Discussion

In this paper, we present an expression and purification protocol for the RBBP6-p53BD with a polyhistidine tag. Furthermore, we report the spectroscopic characterisation of the bacterially expressed RBBP6-p53BD. We also report stability studies of the domain when exposed to an increase in temperature or in the presence of denaturants, which show relatively little structural change in the recombinant domain and a high percentage recovery when returned close to starting condition. Lastly, we showed that the purified RBBP6-p53BD has structure and is functional as it binds endogenous p53.

We evaluated three cell lines for the efficient expression of the recombinant RBBP6-p53BD. Expression levels were found to be similar for NiCo21 (DE3) cells and BL21 (DE3) cells but visibly lower in Shuffle^®^ T7 express (Fig 1). The conditions chosen for future studies were NiCo21 (DE3) cells, at 37°C, 0.1 mM IPTG, and 16 hours post-induction time. The RBBP6-p53BD was found to be expressed in a soluble form under all conditions tested and in all cell lines investigated and produced two protein bands on SDS-PAGE, both at a higher molecular weight than the anticipated 40 KDa. However, a protein migrating to an unexpected distance is not an uncommon phenomenon in SDS-PAGE. This can be due to several factors, including the amino acid residues present in the primary sequence, hydrophobic regions, SDS interaction, and protein structure and stability [32–34, 43]. Protein mobility in an SDS-PAGE relies on the net charge produced by the number of SDS molecules bound to each protein. SDS denatures proteins via its interaction with the protein’s hydrophobic tail and side chains. For highly hydrophilic proteins, SDS is less able to interact and bind to the protein, resulting in aberrant mobility in the gel. ExPASy predicts RBBP6 p53BD to be highly hydrophilic, with a grand average of hydropathicity (GRAVY) of -1.645 [26]. GRAVY values range from 2 to -2, where positive values indicate a protein that is hydrophobic and negative values indicate a hydrophilic protein (S1 Fig). This is further supported by the ExPASy predicted aliphatic index (the relative volume occupied by aliphatic side chains) for the RBBP6 p53BD, which is low at 41.3 [44] (S1 Fig). Aliphatic amino acids are hydrophobic, and therefore proteins that lack them are generally hydrophilic. Interestingly, retarded migration of proteins in SDS-PAGE has been seen for intrinsically disordered proteins. Intrinsically disordered proteins (IDPs) are either unstructured along their entire length or contain significant portions of amino acids that lack structure within the protein [45, 46]. In both predictions using the amino acid sequence (Table 3) and the far-UV CD spectrum (Table 2), we predicted that the RBBP6-p53BD has a large percentage of random coil, suggesting it may be an IDP, or more specifically an intrinsically disordered region (IDR) as it is just a domain found within full length RBBP6.

Looking at the primary amino acid sequence for RBBP6 p53BD, ExPASy calculated RBBP6 p53BD to contain 63.6% amino acid residues promoting disorder and only 19.8% amino acid residues promoting order. This suggests that the domain’s disorder is an intrinsic property derived from its primary amino acid sequence [40, 41]. The UniProtKB database (RBBP6 Unique identifier: Q7Z6E9) predicts high compositional amino acid bias for the residues investigated as the RBBP6 p53BD. Additionally, as mentioned before, the GRAVY score and aliphatic index show that RBBP6 p53BD is highly hydrophilic. This further suggests that RBBP6 p53BD is an intrinsically disordered region, as intrinsically disordered proteins frequently contain biased amino acids, lack hydrophobic amino acid residues and contain a larger portion of disorder promoting amino acid residues [47].

The RBBP6-p53BD was purified out of the soluble fraction of NiCo21 (DE3) cells, to an estimated purity of 95%, in a three-step protocol utilizing hydrophobic interaction chromatography and nickel IMAC chromatography (Fig 3). Two prominent protein bands were present regardless of the chromatography technique used. Often the cause of two or more bands of the same protein appearing in an SDS-PAGE gel is the result of degradation of the protein of interest, which is also a common characteristic of an intrinsically disordered protein (IDP) [45, 46]. However, for the RBBP6-p53BD, the polyhistidine tag was added on the C-terminal end of the recombinant protein, ensuring that only full length recombinant protein would bind the nickel IMAC column [35]. In addition, it was seen in further experiments that when interacting with endogenous p53 (Fig 9) or when run on a native PAGE gel (Fig 4), only a single band could be seen. This suggests that the two protein bands seen in SDS-PAGE are full length, recombinant RBBP6-p53BD and are caused by a reaction in the SDS-PAGE, as on clear native PAGE the protein appears in a single band in the absence of SDS. The clear native PAGE also shows that the RBBP6-p53BD appears in a single form, presumably monomer.

Next, we investigated if the recombinant protein was natively folded and functional. To do this, we performed co-immunoprecipitation assays using endogenous p53 from and purified recombinant RBBP6-p53BD (Fig 9). We found that when antibodies that recognize p53 were used, the RBBP6-p53BD 6 was found in the immuno-complexes eluted, and when an antibody that can bind to the RBBP6-p53BD was used, endogenous p53 was found in the immuno-complexes eluted. This suggests that the recombinant RBBP6-p53BD is folded and functional as it is able to bind to and form a complex with endogenous p53 *in vitro*.

I-TASSER software promotes Model 1 as the most likely conformation. However, Model 2 better matched the DichroWeb predictions made from the far- UV CD spectrum produced from the purified recombinant domain [36]. This is because Model 1 has a much higher beta-strand content than seen present in the far-UV CD spectrum (28% in Model 1 compared to an averaged 9% predicted by DichroWeb). Model 2 shows slightly less structure overall than predicted by DichroWeb but has a similar ratio of alpha-helices to beta-strands (14% alpha helices to 4% beta-strand for Model 2 compared to averaged 19% alpha helices to 9% beta-strand for DichroWeb). The predictions made by Twala investigated a smaller section of full-length RBBP6 as the p53 binding domain, specifically residues 1433–1544 [22]. This is in comparison to residues 1380–1726 investigated in this study. Twala [22] predicted significantly more alpha-helical content than what was predicted across the longer residue sequence in this study by both I-TASSER and DichroWeb. Further studies will be needed to obtain further information on the structure of the domain.

Assessing the stability of a protein is important for evaluating the feasibility of a protein to undergo further testing such as drug interaction studies and structural characterization through X-ray crystallography. Different unfolding methods can result in other unfolding states due to different methods impacting on intra- and intermolecular forces differently. We evaluated the stability of the RBBP6-p53BD using far-UV CD when subjecting the protein to an increase in temperature (Fig 7) and the presence of denaturants (Fig 8).

Thermal unfolding results from the increased energy applied to the protein solution disrupting the stabilising interactions within the protein molecule [48, 49]. When the RBBP6-p53BD was heated to 90°C, the ellipticity became more positive between 200nm and 210nm. However, from 210nm to 250nm, the ellipticity became more negative at higher temperatures, suggesting structural changes depicting a more compact form in parts of the protein (Fig 7A). With thermal unfolding, some native and non-native hydrophobic interactions have been found to be maintained or even strengthened with the increased temperatures [48, 49].

The protein heated to 90°C showed a reasonably small percentage change in structure, ranging between 10 and 30% when different wavelengths were investigated, and significant protein structure remained at 90°C (Fig 7E). Moreover, when the protein was cooled back to 20°C, a good recovery was noted. Approximately 91 to 99.6% recovery was calculated for the different wavelengths for the protein, showing substantial stability when subjected to up to 90°C despite the protein having a predicted high percentage of disorder. This is further seen in the clear native PAGE (Fig 4) as it shows the RBBP6-p53BD, which had been heated to 90°C and cooled back to 20°C was found to migrate the same distance as the sample which was not subjected to the heating process. This suggests similar protein conformations as migration in native PAGE is dependent on the protein’s size, intrinsic charge, and shape [50].

In the presence of urea or guanidinium chloride (Fig 8), the ellipticity of the RBBP6-p53BD increased with an increase in denaturant concentration across the recorded wavelengths. This suggests loss of secondary structure (Fig 8A and 8C). Furthermore, the ellipticity decreased when the denaturant concentration was diluted, suggesting a good recovery of the native structure (Fig 8A and 8C). Exposing the protein to denaturants resulted in substantially more structural change compared to thermal unfolding as above 65–70% structural change was seen in both denaturants compared with a maximum structural change of 30% in thermal unfolding. Also with thermal unfolding, structural changes were seen that suggested the protein became more compact in some parts rather than the structural loss seen in the presence of denaturants. Recovery was also lower in the presence of denaturants compared to thermal unfolding. However, this may be due to the presence of low concentrations of denaturant still being present in the protein sample at the lowest dilution evaluated, and therefore it could not be fully returned to the starting condition like it was in thermal unfolding. It should also be noted that some structure remained even in the presence of 8 M urea or 6 M guanidinium chloride, as the spectrum produced was not completely characteristic of a random coil.

RBBP6-p53BD’s behaviour in response to exposure to denaturants and high temperatures further supports that it is intrinsically disordered. Intrinsically disordered proteins are commonly found to have high resilience to denaturing conditions such as a change in temperature. It has even been seen that some intrinsically disordered proteins appear to gain structure upon exposure to a denaturant, particularly an increase in temperature. This is because intrinsically disordered proteins are often highly hydrophilic. Therefore, when exposed to increasing temperatures, there is an increase in hydrophobic attraction, which is a key force in protein folding [47]. As indicated before, the RBBP6 p53BD is highly hydrophilic; therefore, this could explain why RBBP6 p53BD shows a gain of structure when exposed to increasing temperature. In addition, IDPs can rapidly return to their native state after conditions are reversed. After decreasing the temperature or removing the denaturants, the relatively good structural recovery is another feature consistent with an IDP/IDR. IDPs are frequently found to be resilient under harsh conditions and quickly recover when conditions are reversed.

Additionally, the percentage structural loss is higher and recovery lower for guanidinium chloride compared to urea, suggesting it has a stronger denaturing effect on the RBBP6-p53BD. However, the exact molecular mechanism of denaturation for either guanidinium chloride or urea is still not fully known. There are several reasons why one denaturant may be more effective than another on a specific protein. The two leading ideas are the actual interaction of the denaturant with the protein and the modification of the protein’s environment, which then disrupts hydrophobic interactions within the protein [51, 52]. Urea is known to form hydrogen bonds with the peptide backbone, disrupting the stabilizing interactions within the protein [53]. Guanidinium chloride disrupts hydrophobic interactions in the peptide backbone and aromatic side chains [54]. Another noteworthy difference seen is that urea seems to first destabilize beta-strands, and guanidinium chloride seems to first destabilize alpha-helices [52]. Therefore, proteins with a higher alpha-helical content are more readily unfolded by guanidinium chloride than urea. This is significant as using the far-UV CD spectrum, DICHROWEB [36] predicted that the RBBP6-p53BD has more than double the alpha-helical content than beta-strand content (Table 2) and further supports I-TASSER model 2 as the better prediction.

## 5. Conclusion

We have successfully expressed a functional RBBP6-p53BD and characterized its secondary structure. The recombinant protein was successfully purified to approximately 95% homogeneity, using a novel three-step purification protocol. The recombinant protein can bind endogenous p53 indicating an essential property for downstream uses to describe the mechanism of action of p53 and RBBP6. It is critical to understand the essential features of the p53-Mdm2-RBBP6 interactions. Since this domain is potentially druggable, the recombinant protein is vital for drug discovery and development.

## Supporting information

S1 Fig ExPASy results for RBBP6 p53BD sequence. Results produced by ExPASy using the RBBP6 p53BD amino acid sequence investigated in this study. Including the amino acid sequence submitted, theoretical pI, the extinction coefficient, instability index, aliphatic index and GRAVY scores.
(PDF)

S1 Raw images (PDF)

## References

[1] Pardee AB. G1 events and regulation of cell proliferation. Science. 1989;246(4930):603–8. doi: 10.1126/science.2683075 2683075

[2] Hartwell LH, Weinert TA. Checkpoints: controls that ensure the order of cell cycle events. Science. 1989;246(4930):629–34. doi: 10.1126/science.2683079 2683079

[3] Haupt Y, Maya R, Kazaz A, Oren M. Mdm2 promotes the rapid degradation of p53. Nature. 1997;387(6630):296–9. doi: 10.1038/387296a0 9153395

[4] Poyurovsky MV, Katz C, Laptenko O, Beckerman R, Lokshin M, Ahn J, et al. The C terminus of p53 binds the N-terminal domain of MDM2. Nat Struct Mol Biol. 2010;17(8):982–9. doi: 10.1038/nsmb.1872 20639885 PMC2922928

[5] Honda R, Tanaka H, Yasuda H. Oncoprotein MDM2 is a ubiquitin ligase E3 for tumor suppressor p53. FEBS Lett. 1997;420(1):25–7. doi: 10.1016/s0014-5793(97)01480-4 9450543

[6] Li L, Deng B, Xing G, Teng Y, Tian C, Cheng X, et al. PACT is a negative regulator of p53 and essential for cell growth and embryonic development. Proc Natl Acad Sci U S A. 2007;104(19):7951–6. doi: 10.1073/pnas.0701916104 17470788 PMC1876553

[7] Momand J, Zambetti GP, Olson DC, George D, Levine AJ. The mdm-2 oncogene product forms a complex with the p53 protein and inhibits p53-mediated transactivation. Cell. 1992;69(7):1237–45. doi: 10.1016/0092-8674(92)90644-r 1535557

[8] Oliner JD, Pietenpol JA, Thiagalingam S, Gyuris J, Kinzler KW, Vogelstein B. Oncoprotein MDM2 conceals the activation domain of tumour suppressor p53. Nature. 1993;362(6423):857–60. doi: 10.1038/362857a0 8479525

[9] Barak Y, Juven T, Haffner R, Oren M. mdm2 expression is induced by wild type p53 activity. Embo j. 1993;12(2):461–8. doi: 10.1002/j.1460-2075.1993.tb05678.x 8440237 PMC413229

[10] Simons A, Melamed-Bessudo C, Wolkowicz R, Sperling J, Sperling R, Eisenbach L, et al. PACT: cloning and characterization of a cellular p53 binding protein that interacts with Rb. Oncogene. 1997;14(2):145–55. doi: 10.1038/sj.onc.1200825 9010216

[11] Kappo MA, Ab E, Hassem F, Atkinson RA, Faro A, Muleya V, et al. Solution structure of RING finger-like domain of retinoblastoma-binding protein-6 (RBBP6) suggests it functions as a U-box. J Biol Chem. 2012;287(10):7146–58. doi: 10.1074/jbc.M110.217059 22130672 PMC3293548

[12] Di Giammartino DC, Li W, Ogami K, Yashinskie JJ, Hoque M, Tian B, et al. RBBP6 isoforms regulate the human polyadenylation machinery and modulate expression of mRNAs with AU-rich 3’ UTRs. Genes Dev. 2014;28(20):2248–60. doi: 10.1101/gad.245787.114 25319826 PMC4201286

[13] Chen J, Tang H, Wu Z, Zhou C, Jiang T, Xue Y, et al. Overexpression of RBBP6, alone or combined with mutant TP53, is predictive of poor prognosis in colon cancer. PLoS One. 2013;8(6):e66524. doi: 10.1371/journal.pone.0066524 23799110 PMC3684577

[14] Moela P, Motadi LR. RBBP6: a potential biomarker of apoptosis induction in human cervical cancer cell lines. Onco Targets Ther. 2016;9:4721–35. doi: 10.2147/OTT.S100964 27536134 PMC4973719

[15] Mbita Z, Hull R, Mbele M, Makhafola T, Dlamini Z. Expression Analysis of RbBP6 in human cancers: a Prospective biomarker. Anticancer Drugs. 2019;30(8):767–73. doi: 10.1097/CAD.0000000000000809 31274515

[16] Xiao C, Wu G, Zhou Z, Zhang X, Wang Y, Song G, et al. RBBP6, a RING finger-domain E3 ubiquitin ligase, induces epithelial-mesenchymal transition and promotes metastasis of colorectal cancer. Cell Death Dis. 2019;10(11):833. doi: 10.1038/s41419-019-2070-7 31685801 PMC6828677

[17] Wang QS, Wei SR, Xiao HL. RBBP6 induces non-small cell lung cancer cell proliferation and high expression is associated with poor prognosis. Oncol Lett. 2020;19(4):2895–901. doi: 10.3892/ol.2020.11403 32218844 PMC7068609

[18] Ntwasa M, Nweke E, and Cajee U. The Retinoblastoma Binding Protein 6 Family is Essential for Embryonic Development and Carcinogenesis. Journal of Cancer Research Forecast. 2018;1:1002.

[19] Gao S, Scott RE. Stable overexpression of specific segments of the P2P-R protein in human MCF-7 cells promotes camptothecin-induced apoptosis. J Cell Physiol. 2003;197(3):445–52. doi: 10.1002/jcp.10381 14566974

[20] Ndabambi N. Recombinant expression of the pRb- and p53-interacting domains from the human RBBP6 protein for in vitro binding studies.: University of the Western Cape; 2004.

[21] Faro A. Investigation of the interactions of Retinoblastoma Binding Protein-6 with transcription factors p53 and Y-Box Binding Protein-1.: University of the Western Cape; 2011.

[22] Twala C. Drugs targeting the retinoblastoma binding protein 6 (RBBP6). University of the Witwatersrand; 2017.

[23] Shevchenko A, Tomas H, Havlis J, Olsen JV, Mann M. In-gel digestion for mass spectrometric characterization of proteins and proteomes. Nat Protoc. 2006;1(6):2856–60. doi: 10.1038/nprot.2006.468 17406544

[24] Roth R, van Zyl P, Tsekoa T, Stoychev S, Mamputha S, Buthelezi S, et al. Co-expression of sulphydryl oxidase and protein disulphide isomerase in Escherichia coli allows for production of soluble CRM(197). J Appl Microbiol. 2017;122(5):1402–11. doi: 10.1111/jam.13441 28276616

[25] McLellan T. Electrophoresis buffers for polyacrylamide gels at various pH. Anal Biochem. 1982;126(1):94–9. doi: 10.1016/0003-2697(82)90113-0 7181120

[26] Wilkins MR, Gasteiger E, Bairoch A, Sanchez J-C, Williams KL, Appel RD, et al. Protein Identification and Analysis Tools in the ExPASy Server. In: Link AJ, editor. 2-D Proteome Analysis Protocols. Totowa, NJ: Humana Press; 1999. p. 531–52. 10.1385/1-59259-584-7:531 10027275

[27] Pace CN. Determination and analysis of urea and guanidine hydrochloride denaturation curves. Methods Enzymol. 1986;131:266–80. doi: 10.1016/0076-6879(86)31045-0 3773761

[28] Studier FW, Moffatt BA. Use of bacteriophage T7 RNA polymerase to direct selective high-level expression of cloned genes. J Mol Biol. 1986;189(1):113–30. doi: 10.1016/0022-2836(86)90385-2 3537305

[29] Robichon C, Luo J, Causey TB, Benner JS, Samuelson JC. Engineering Escherichia coli BL21(DE3) derivative strains to minimize E. coli protein contamination after purification by immobilized metal affinity chromatography. Appl Environ Microbiol. 2011;77(13):4634–46. doi: 10.1128/AEM.00119-11 21602383 PMC3127686

[30] Lobstein J, Emrich CA, Jeans C, Faulkner M, Riggs P, Berkmen M. SHuffle, a novel Escherichia coli protein expression strain capable of correctly folding disulfide bonded proteins in its cytoplasm. Microb Cell Fact. 2012;11:56. doi: 10.1186/1475-2859-11-56 22569138 PMC3526497

[31] Shoae M, Safarpour H, Khorashadizadeh M. Recombinant Production of Bovine Enteropeptidase Light Chain in SHuffle^®^ T7 Express and Optimization of Induction Parameters. Protein J. 2021;40(6):907–16. 34586553 10.1007/s10930-021-10022-9

[32] Banker GA, Cotman CW. Measurement of free electrophoretic mobility and retardation coefficient of protein-sodium dodecyl sulfate complexes by gel electrophoresis. A method to validate molecular weight estimates. J Biol Chem. 1972;247(18):5856–61. 4560419

[33] Rath A, Cunningham F, Deber CM. Acrylamide concentration determines the direction and magnitude of helical membrane protein gel shifts. Proc Natl Acad Sci U S A. 2013;110(39):15668–73. doi: 10.1073/pnas.1311305110 24019476 PMC3785759

[34] Yarawsky AE, English LR, Whitten ST, Herr AB. The Proline/Glycine-Rich Region of the Biofilm Adhesion Protein Aap Forms an Extended Stalk that Resists Compaction. J Mol Biol. 2017;429(2):261–79. doi: 10.1016/j.jmb.2016.11.017 27890783 PMC5363081

[35] Eschenfeldt WH, Maltseva N, Stols L, Donnelly MI, Gu M, Nocek B, et al. Cleavable C-terminal His-tag vectors for structure determination. J Struct Funct Genomics. 2010;11(1):31–9. doi: 10.1007/s10969-010-9082-y 20213425 PMC2885959

[36] Miles AJ, Ramalli SG, Wallace BA. DichroWeb, a website for calculating protein secondary structure from circular dichroism spectroscopic data. Protein Sci. 2021. doi: 10.1002/pro.4153 34216059 PMC8740839

[37] Wu S, Zhang Y. LOMETS: a local meta-threading-server for protein structure prediction. Nucleic Acids Res. 2007;35(10):3375–82. doi: 10.1093/nar/gkm251 17478507 PMC1904280

[38] Roy A, Kucukural A, Zhang Y. I-TASSER: a unified platform for automated protein structure and function prediction. Nat Protoc. 2010;5(4):725–38. doi: 10.1038/nprot.2010.5 20360767 PMC2849174

[39] Klose DP, Wallace BA, Janes RW. 2Struc: the secondary structure server. Bioinformatics. 2010;26(20):2624–5. doi: 10.1093/bioinformatics/btq480 20739308 PMC2951091

[40] Dunker AK, Lawson JD, Brown CJ, Williams RM, Romero P, Oh JS, et al. Intrinsically disordered protein. J Mol Graph Model. 2001;19(1):26–59. doi: 10.1016/s1093-3263(00)00138-8 11381529

[41] Dyson HJ, Wright PE. Intrinsically unstructured proteins and their functions. Nat Rev Mol Cell Biol. 2005;6(3):197–208. doi: 10.1038/nrm1589 15738986

[42] Ranjbar B, Gill P. Circular dichroism techniques: biomolecular and nanostructural analyses- a review. Chem Biol Drug Des. 2009;74(2):101–20. doi: 10.1111/j.1747-0285.2009.00847.x 19566697

[43] Rath A, Glibowicka M, Nadeau VG, Chen G, Deber CM. Detergent binding explains anomalous SDS-PAGE migration of membrane proteins. Proc Natl Acad Sci U S A. 2009;106(6):1760–5. doi: 10.1073/pnas.0813167106 19181854 PMC2644111

[44] Ikai A. Thermostability and aliphatic index of globular proteins. J Biochem. 1980;88(6):1895–8. 7462208

[45] Tompa P. Intrinsically unstructured proteins evolve by repeat expansion. Bioessays. 2003;25(9):847–55. doi: 10.1002/bies.10324 12938174

[46] Uversky VN. The mysterious unfoldome: structureless, underappreciated, yet vital part of any given proteome. J Biomed Biotechnol. 2010;2010:568068. doi: 10.1155/2010/568068 20011072 PMC2789583

[47] Uversky VN. Paradoxes and wonders of intrinsic disorder: Stability of instability. Intrinsically Disord Proteins. 2017;5(1):e1327757. doi: 10.1080/21690707.2017.1327757 30250771 PMC6149434

[48] Baldwin RL. Temperature dependence of the hydrophobic interaction in protein folding. Proc Natl Acad Sci U S A. 1986;83(21):8069–72. doi: 10.1073/pnas.83.21.8069 3464944 PMC386868

[49] Bryngelson JD, Onuchic JN, Socci ND, Wolynes PG. Funnels, pathways, and the energy landscape of protein folding: a synthesis. Proteins. 1995;21(3):167–95. doi: 10.1002/prot.340210302 7784423

[50] Schägger H, Cramer WA, von Jagow G. Analysis of molecular masses and oligomeric states of protein complexes by blue native electrophoresis and isolation of membrane protein complexes by two-dimensional native electrophoresis. Anal Biochem. 1994;217(2):220–30. doi: 10.1006/abio.1994.1112 8203750

[51] Frank HS, Franks F. Structural Approach to the Solvent Power of Water for Hydrocarbons; Urea as a Structure Breaker. Journal of Chemical Physics. 1968;48:4746–57.

[52] Camilloni C, Rocco AG, Eberini I, Gianazza E, Broglia RA, Tiana G. Urea and guanidinium chloride denature protein L in different ways in molecular dynamics simulations. Biophys J. 2008;94(12):4654–61. doi: 10.1529/biophysj.107.125799 18339753 PMC2397339

[53] Candotti M, Esteban-Martín S, Salvatella X, Orozco M. Toward an atomistic description of the urea-denatured state of proteins. Proc Natl Acad Sci U S A. 2013;110(15):5933–8. doi: 10.1073/pnas.1216589110 23536295 PMC3625277

[54] Parui S, Manna RN, Jana B. Destabilization of Hydrophobic Core of Chicken Villin Headpiece in Guanidinium Chloride Induced Denaturation: Hint of π-Cation Interaction. J Phys Chem B. 2016;120(36):9599–607. 27548328 10.1021/acs.jpcb.6b06325

