# Supplementary material for: Expression, purification, and characterisation of the p53 binding domain of Retinoblastoma binding protein 6 (RBBP6)
Source: PLoS One. 2023 Feb 10;18(2):e0277478. doi: 10.1371/journal.pone.0277478 (PMC9916574; doi:10.1371/journal.pone.0277478)
Supplement: S1 Fig — Results produced by ExPASy using the RBBP6 p53BD amino acid sequence investigated in this study. Including the amino acid sequence submitted, theoretical pI, the extinction coefficient, instability index, aliphatic index and GRAVY scores. (PDF) [file pone.0277478.s001.pdf]

```
        10         20         30         40         50         60
VSHEIIQHEV KSSKNSASSE KGKTKDRDYS VLEKENPEKR KNSTQPEKES NLDRLNEQGN

        70         80         90        100        110        120
FKSLSQSSKE ARTSDKHDST RASSNKDFTP NRDKKTDYDT REYSSSKRRD EKNELTRRKD

       130        140        150        160        170        180
SPSRNKDSAS GQKNKPREER DLPKKGTGDS KKSNSSPSRD RKPHDHKATY DTKRPNEETK

       190        200        210        220        230        240
SVDKNPCKDR EKHVLEARNN KESSGNKLLY ILNPPETQVE KEQITGQIDK STVKPKPQLS

       250        260        270        280        290        300
HSSRLSSDLT RETDEAAFEP DYNESDSESN VSVKEEESSG NISKDLKDKI VEKAKESLDT

       310        320        330        340
AAVVQVGISR NQSHSSPSVS PSRSHSPSGS QTRSHSSSAS SAESQDS
```

Total number of negatively charged residues (Asp + Glu): 62
Total number of positively charged residues (Arg + Lys): 68

Atomic composition:

Carbon	C	1615
Hydrogen	H	2646
Nitrogen	N	518
Oxygen	O	597
Sulfur	S	1

Number of amino acids: 347

Molecular weight: 38903.95

Theoretical pI: 9.00

Formula: $C_{1615}H_{2646}N_{518}O_{597}S_{1}$
Total number of atoms: 5377

Extinction coefficients:

This protein does not contain any Trp residues. Experience shows that this could result in more than 10% error in the computed extinction coefficient.

Extinction coefficients are in units of $M^{-1} cm^{-1}$, at 280 nm measured in water.

Ext. coefficient 8940
Abs 0.1% (=1 g/l) 0.230, assuming all pairs of Cys residues form cystines

Ext. coefficient 8940
Abs 0.1% (=1 g/l) 0.230, assuming all Cys residues are reduced

Estimated half-life:

The N-terminal of the sequence considered is V (Val).

The estimated half-life is: 100 hours (mammalian reticulocytes, in vitro).
>20 hours (yeast, in vivo).
>10 hours (Escherichia coli, in vivo).

Instability index:

The instability index (II) is computed to be 58.83
This classifies the protein as unstable.

Aliphatic index: 41.30

Grand average of hydropathicity (GRAVY): -1.645

S1 Fig. ExPASy results for RBBP6 p53BD sequence.

Results produced by ExPASy using the RBBP6 p53BD amino acid sequence investigated in this study. Including the amino acid sequence submitted, theoretical pI, the extinction coefficient, instability index, aliphatic index and GRAVY scores.
