# Supplementary material for: Expression, purification, and characterisation of the p53 binding domain of Retinoblastoma binding protein 6 (RBBP6)
Source: PLoS One. 2023 Feb 10;18(2):e0277478. doi: 10.1371/journal.pone.0277478 (PMC9916574; doi:10.1371/journal.pone.0277478)
Supplement: S1 Raw images — (PDF) [file pone.0277478.s002.pdf]

Figure 1 A

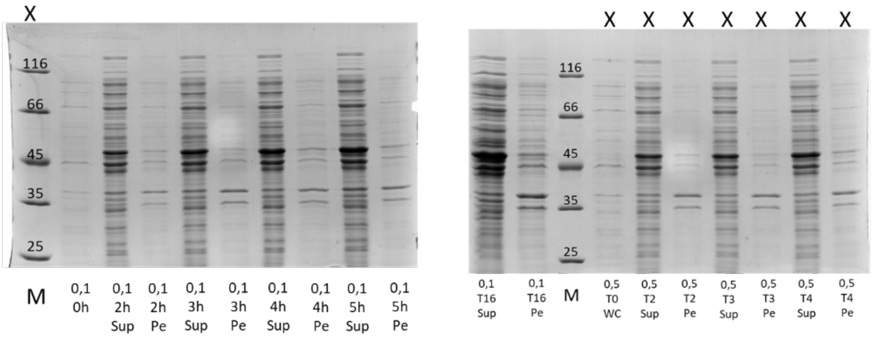

Figure 1 B

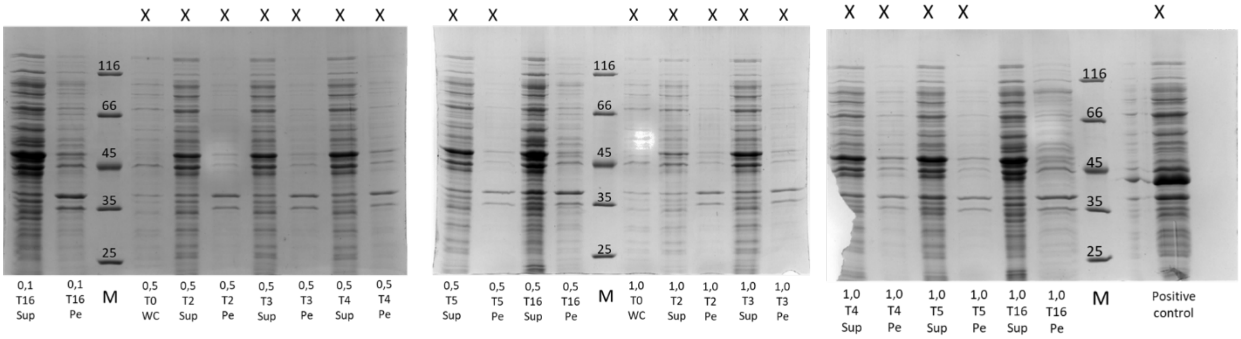

Fig 1A and B raw images

SDS-PAGE analysis of expression conditions. IPTG concentration is shown (0.1, 0.5 or 1.0mM), as well as post induction time ("X"h or T"X"). Soluble "Sup" and insoluble "Pe" fractions are indicated. Lanes not included in final image are marked with an X above them. For (A) Expression from 0 to 16 hours post induction for BL21 (DE3) cells induced with 0.1mM IPTG. In addition showing 0, 2, 3 and 4 hour post induction for 0.5mM IPTG not include in final image. (B) From three separate gels, T16 (16 hours post induction time) samples were taken and compared for 3 IPTG concentrations (0.1, 0.5 and 1.0mM) for BL21 (DE3) cells, other times not shown in final image. Molecular weight makers indicated (M), with molecular weight of bands marked on gel.

Figure 1 C

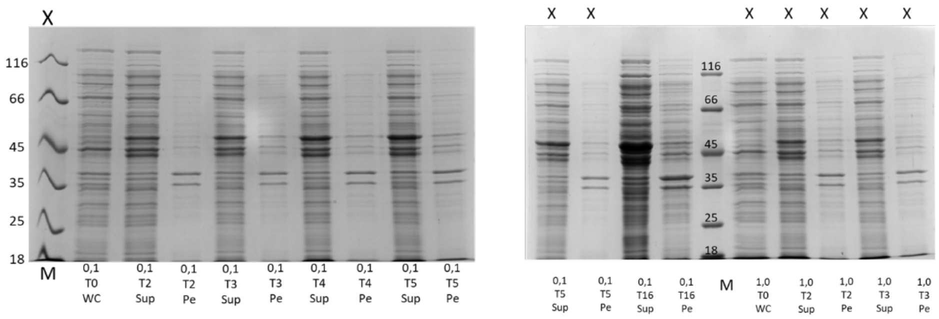

Figure 1 D

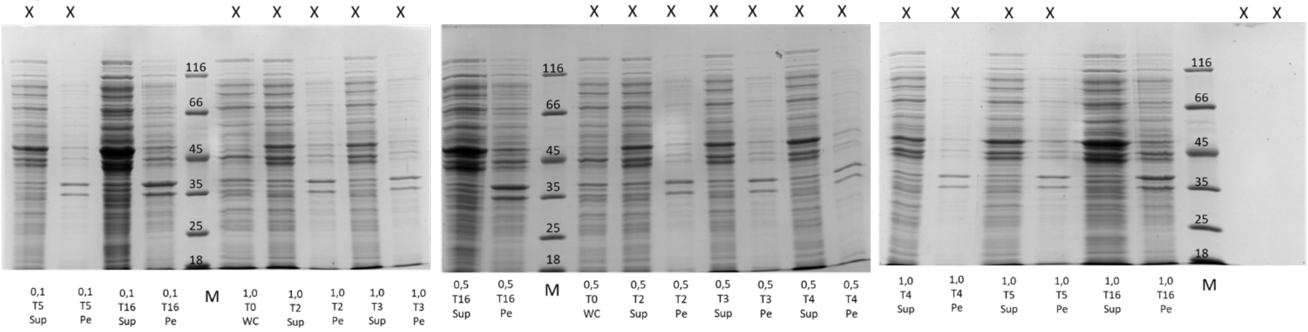

Fig 1C and D raw images

SDS-PAGE analysis of expression conditions. IPTG concentration is shown (0.1, 0.5 or 1.0mM), as well as post induction time (Xh or TX). Soluble “Sup” and insoluble “Pe” fractions are indicated. Lanes not included in final image are marked with an X above them. For (C) Expression from 0 to 16 hours post induction for NiCo21 (DE3) cells induced with 0.1mM IPTG. In addition showing 0, 2, 3 and 4 hour post induction for 0.5mM IPTG not include in final image. (D) From three separate gels, T16 (16 hours post induction) samples were taken and compared for 3 IPTG concentrations (0.1, 0.5 and 1.0mM) for NiCo21 (DE3) cells, other times not shown in final image. Molecular weight makers indicated (M), with molecular weight of bands marked on gel.

Figure 1 E

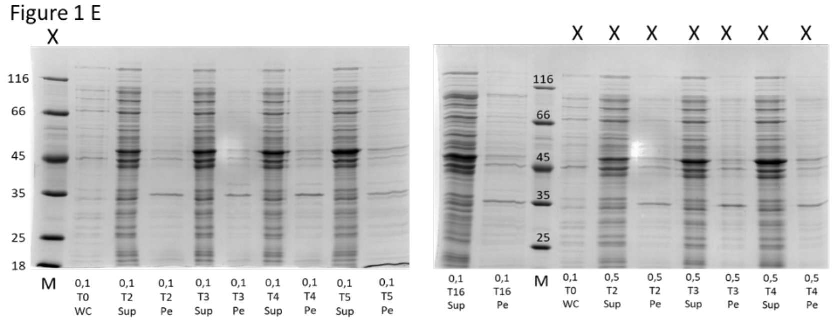

Figure 1 F

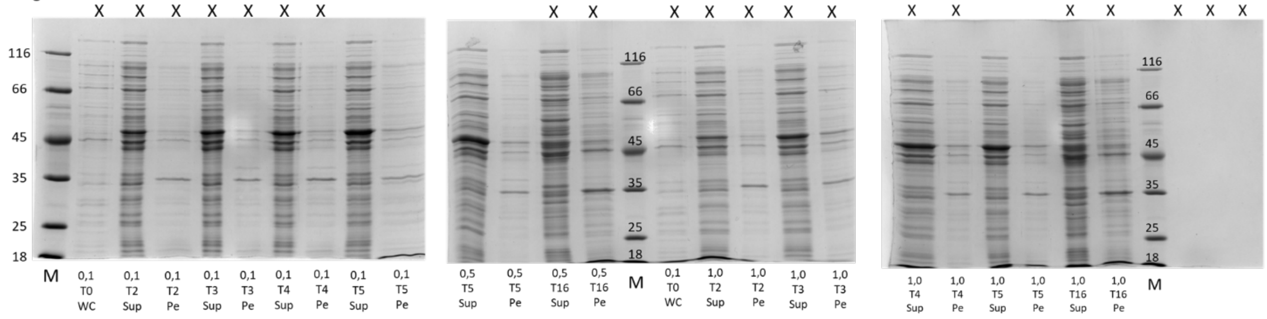

Fig 1E and F raw images

SDS-PAGE analysis of expression conditions. IPTG concentration is shown (0.1, 0.5 or 1.0mM), as well as post induction time (Xh or TX). Soluble "Sup" and insoluble "Pe" fractions are indicated. Lanes not included in final image are marked with an X above them. For (E) Expression from 0 to 16 hours post induction for Shuffle ® T7 express cells induced with 0.1mM IPTG. In addition showing 0, 2, 3 and 4 hour post induction for 0.5mM IPTG not include in final image. (F) From three separate gels, T5 (5 hours post induction) samples were taken and compared for 3 IPTG concentrations (0.1, 0.5 and 1.0mM) for Shuffle ® T7 express cells, other times not shown in final image. Molecular weight makers indicated (M) with molecular weight of bands marked on gel.

Figure 2 A

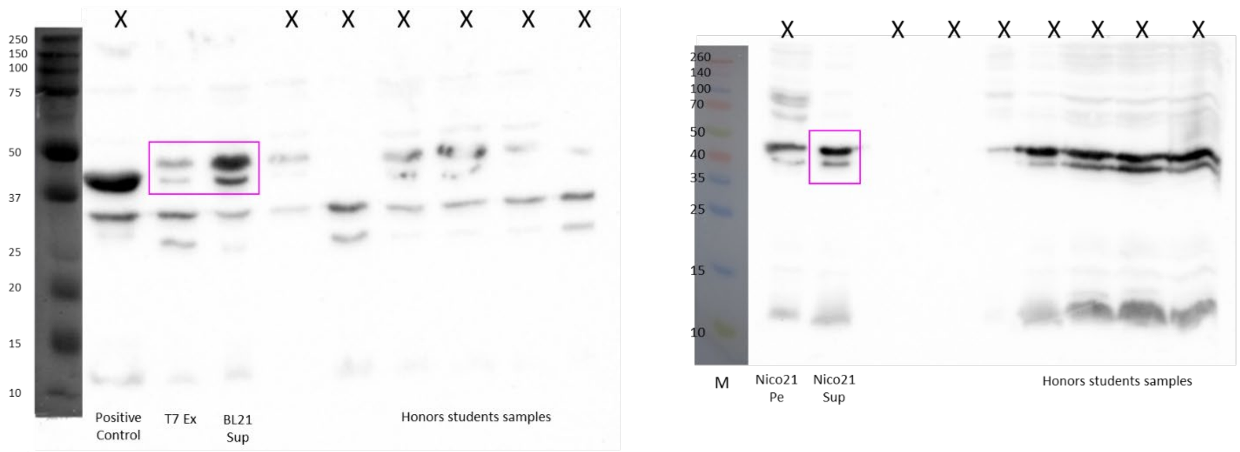

Figure 2 B

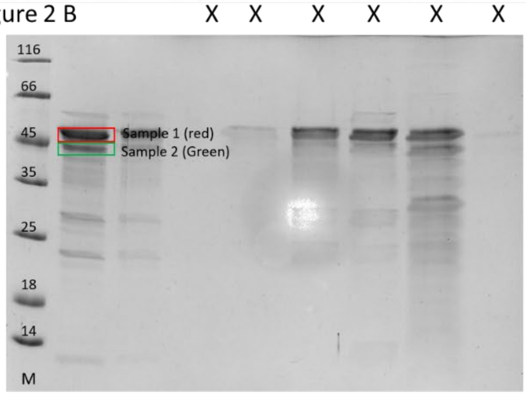

Fig 2 A and B raw images

(A)Western blots preformed to confirm the presence of a polyhistidine tag in overexpressed bands in the supernatant samples of Shuffle® T7 express and BL21 (DE3) cells in the first blot (left) and NiCo21 (DE3) cells in the second blot (right). Multiple honours student's samples from their expression studies were run on the same blot and are not included in the final image. The molecular weight markers were used to stain the membrane and then sized to blot images. Molecular weights of the protein bands in the marker (M) are indicated on the blots. (B) The SDS-PAGE that had bands excised to be sent for mass spectrometry analysis. The two bands excided are indicated and then additional lanes show older samples of fractions collected during purification trials. The smeared effect on right hand side of gel is due to ammonium sulphate being present in the samples.

Figure 3 D

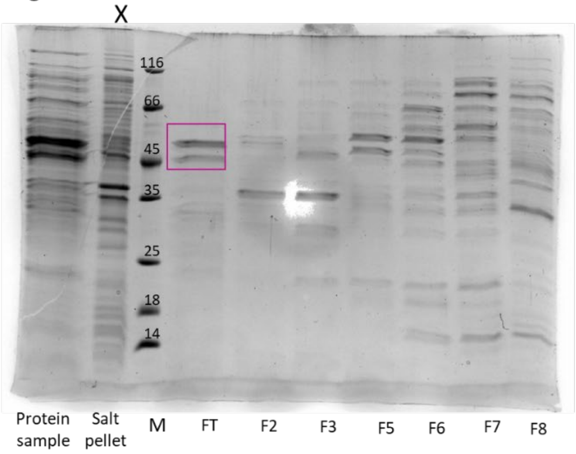

Figure 3 D continued

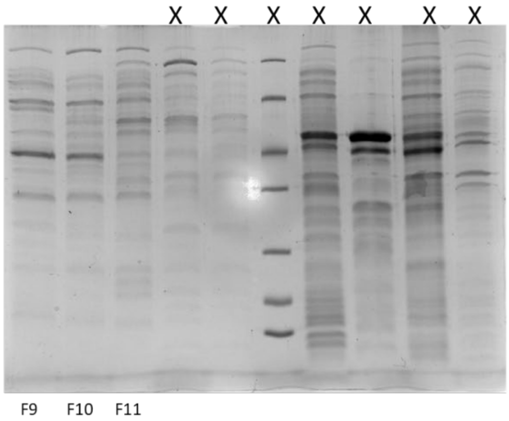

Figure 3 E

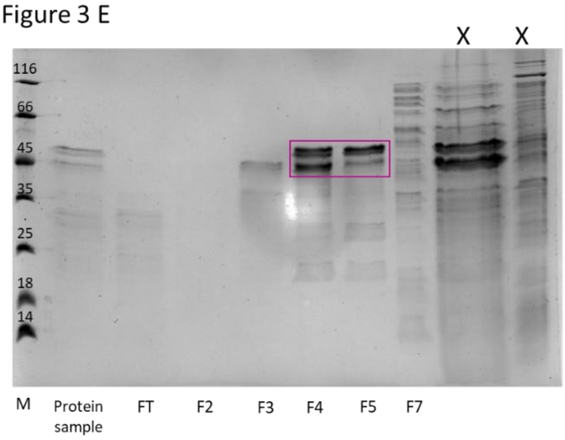

Figure 3 F

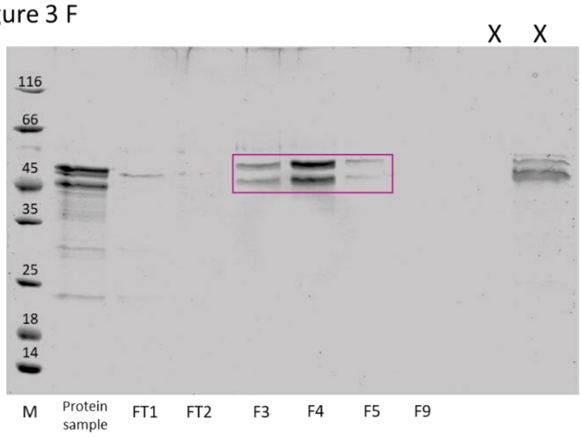

Fig 3 D, E and F raw images

(D) SDS-PAGE gel showing samples used and collected during the first part of purification of the soluble fraction of NICO21 (DE3) cells (1M ammonium sulphate using a phenol column). Lanes not included in final image are indicated by an X above the gel. The salt pellet, is the proteins precipitated out of solution and collected are centrifugation of the supernatant sample after incubation in 1M ammonium sulphate. (E) SDS-PAGE analysis of samples used and collected during the second part of purification protocol (1.4M ammonium sulphate using a phenol column). Protein bands are smeared due to the presence of high concentration of ammonium sulphate in the samples. Lanes not included in final image are indicated by an X above the gel, and aren't relevant to the purification being analysed. (F) SDS-PAGE gel showing samples used in and collected during the third part of purification (Nickel affinity chromatography). Lanes not included in final image are indicated by an X above the gel, final lane is the protein sample before dialysis (to remove ammonium sulphate). Molecular weight makers indicated (M), with molecular weight of bands marked on gel.

Figure 4 A

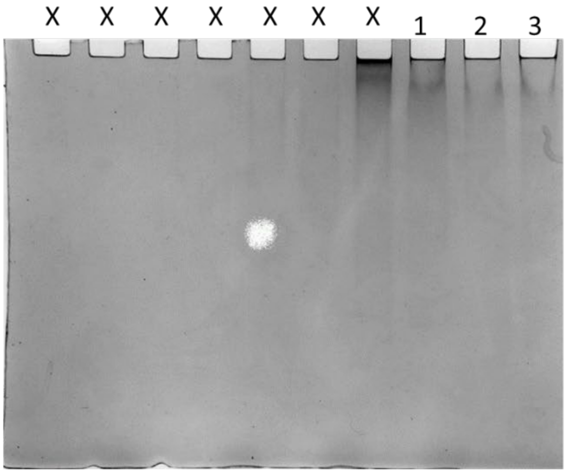

Figure 4 B

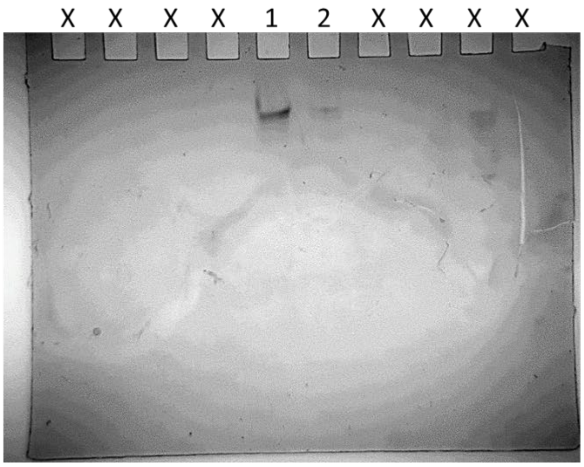

Fig 4 A and B raw images

Full gels showing RBBP6 p53BD samples loaded onto (A) Tris-HCl native PAGE gel at pH 8.3 and (B) HEPES-Imidazole native PAGE at pH 7.4. Lanes not included in final image are marked with an X above them. Electrodes were reversed as RBBP6 p53BD has an estimated pI of 9.

Figure 9

Anti-p53 WB

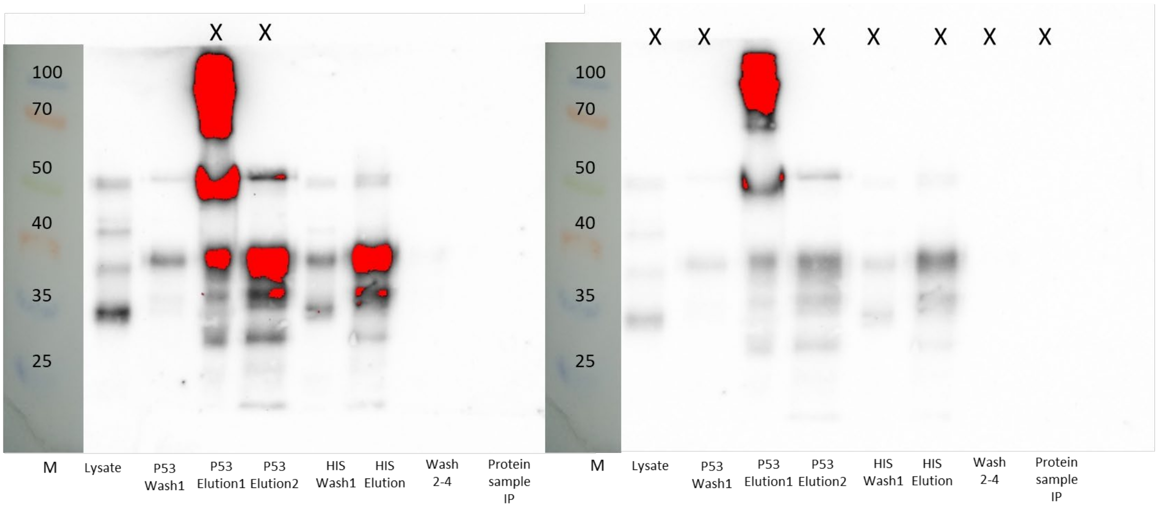

Anti-polyhistidine WB

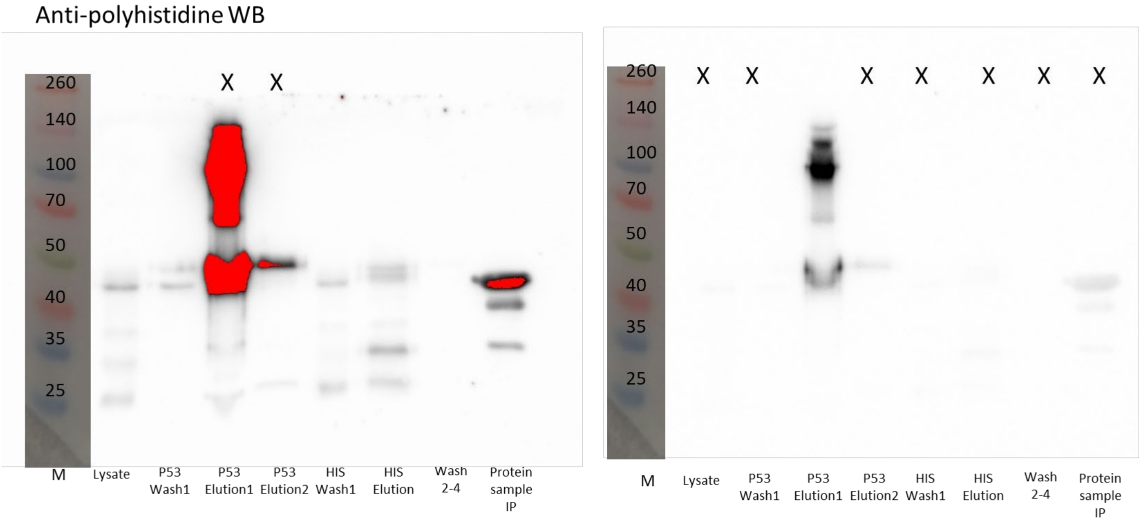

Fig 9 raw images

Full Western blots images for p53 and polyhistidine. The molecular weight markers were used to stain the membrane and then sized to blot images. Two exposure levels were used as p53 elution 1 lane was overexposed when other lanes were visible. In p53 elution lane, you can see a band at approximately 50 kDa and 25Kda, this represents the remaining antibody chain's removed with the reducing sample buffer elution step, this was not included in final image, and is marked with an X in all blots. P53 elution 1 shows p53 at approximately 50KDa, and a band around 150kDa of non-reduced antibodies. The presence of the antibody chains in the western blot images is due to the same species (mouse) generating the antibodies used in the Co-IP assays as well as the Western blot assay. The anti-polyhistidine blots show bands at 50kDa and 25KDa, indicating the antibody heavy and light chains, as the anti-polyhistidine antibody was also raised in mice. Furthermore the His elution lane shows a band at approximately 50 kDa and 25KDa, showing the heavy and light antibody chains in addition to a band around 48kDa, representing the polyhistidine tagged RBBP6 p53BD. This is because a non-reducing elution step was not performed for the anti-polyhistidine Co-IP assay.
